# Architecture-Dependent Thermal Decomposition of RAFT-Modified Polypropylene Glycol Maleate-Acrylic Acid Copolymers: Results of TG–MS and Kinetic Analysis

**DOI:** 10.3390/polym18131599

**Published:** 2026-06-26

**Authors:** Akmaral Zh. Sarsenbekova, Almagul S. Makhmutova, Meruyert S. Zhunissova, Nazigul S. Remetova, Meruyert B. Issabayeva, Gulnissa K. Kurmantayeva, Mussa E. Zholdasbayev, Bibigul B. Ashirbekova

**Affiliations:** 1 Chemistry Faculty, Karaganda Buketov National Research University, Karaganda 100024, Kazakhstan; chem_akmaral@mail.ru; 2 School of Pharmacy, Karaganda Medical University, Karaganda 100024, Kazakhstan

**Keywords:** RAFT polymerization, polypropylene glycol maleate, acrylic acid copolymers, macromolecular architecture, alkaline hydrolysis, gel permeation chromatography, TG–MS, thermal degradation, isoconversional kinetics, density functional theory

## Abstract

The effect of reversible addition–fragmentation chain transfer (RAFT) polymerization on the structure, morphology, and thermal degradation behavior of polypropylene glycol maleate–acrylic acid copolymers (p-PGM:AA) was investigated using 2-cyano-2-propyl dodecyl trithiocarbonate (CPDT) as the RAFT agent. Copolymers synthesized at different CPDT concentrations were characterized by ^1^H/^13^C NMR spectroscopy, gel permeation chromatography (GPC), transmission electron microscopy (TEM), thermogravimetric analysis coupled with mass spectrometry (TG–MS), isoconversional kinetic methods, and density functional theory (DFT) calculations. ^1^H NMR spectroscopy revealed a progressive decrease in the relative intensity of vinyl proton signals with increasing CPDT concentration, indicating enhanced conversion of unsaturated fragments during copolymerization. Alkaline hydrolysis followed by ^1^H NMR and GPC analysis of the degradation products confirmed cleavage of polyester segments and yielded low-molecular-weight fragments with *M_n_* = 1370 g mol^−1^ and narrow dispersity (*Đ* = 1.035), providing additional information on the architecture of the vinyl-polymerized segments. Increasing CPDT concentration resulted in lower molecular weights and narrower molecular weight distributions of the soluble copolymer fractions. TEM analysis demonstrated broader domain size distributions and increased morphological heterogeneity in RAFT-modified samples, accompanied by an increase in swelling degree. Thermogravimetric analysis showed that RAFT-modified systems undergo multi-stage thermal degradation with the appearance of an additional low-temperature stage associated with thermolabile fragments. TG–MS revealed earlier evolution of CO_2_ and oxygen-containing species and changes in the distribution of volatile products. DFT calculations indicated a decrease in the HOMO–LUMO energy gap and suggested the participation of RAFT-derived fragments in the energetic characteristics of decarboxylation processes. Isoconversional and nonlinear kinetic analyses demonstrated increased kinetic heterogeneity for branched copolymer s synthesized at elevated CPDT concentrations, whereas cross-linked systems exhibited more uniform degradation behavior. The combined experimental and theoretical results demonstrate that RAFT polymerization provides an effective route for tuning the macromolecular architecture, morphology, and thermal degradation pathways of p-PGM:AA copolymers.

## 1. Introduction

Controlled radical polymerization is currently considered as one of the key approaches to the synthesis of polymers with a given macro-molecular architecture and regulated properties [1,2,3,4,5,6,7,8,9,10,11,12]. Among the existing methods, a special place is occupied by polymerization with reversible addition–fragmentation chain transfer (RAFT) mechanism, which provides the ability to purposefully control the growth of chains, their branching and the formation of macromolecules of various topologies [3,4,5,6,7,8,9,10,11,12].

The RAFT polymerization mechanism is based on degenerative chain transfer, in which a dynamic equilibrium is established between active radical centres and ‘dormant’ forms [3,4,5,6,7,8,9,10,11,12]. This allows for control over the growth and structure of macromolecules, as well as ensuring high tolerance to functional monomers, including acrylic acid [7,8,9].

Copolymerization of polypropylene glycol maleate (p-PGM) with acrylic acid (AA) under the conditions of traditional radical polymerization leads to the formation of systems with an uncontrolled architecture, including linear, branched and spatially cross-linked structures [13,14]. The properties of such materials depend on both the distribution of functional groups and the spatial organization of macromolecules, while the presence of carboxyl groups contributes to their swelling behavior [7,9,13]. The p-PGM:AA system is of interest due to the combination of unsaturated polyester fragments and reactive carboxyl groups, which opens up opportunities for the production of materials with controlled characteristics [7,13,14].

The use of RAFT polymerization in this system allows variation in the chain length and degree of branching of macromolecules [7,8,11]. An increase in the concentration of RAFT agent is accompanied by a reduction in the chain length, a change in the architecture and redistribution of functional groups, which affects the morphology (size and distribution of domains) and physicochemical properties of materials [1,3,8,11].

Despite the significant amount of work on RAFT polymerization of acrylic systems, research is mainly focused on soluble macromolecules [1,9,11].

The unsaturated polyester resins based on polypropylene glycol maleate (p-PGM) investigated in this work [15,16] represent a promising model system for studying architecture-dependent thermal degradation. Their structure combines flexible polyester segments, reactive maleate double bonds, and thermosensitive ester groups within a single macromolecular framework. In contrast to conventionally studied linear or predominantly soluble acrylic RAFT systems, p-PGM-based copolymers are characterized by the ability to form branched and partially cross-linked structures with heterogeneous macromolecular organization.

The presence of unsaturated maleate fragments provides the possibility for RAFT-modified copolymerization, whereas the polyester backbone determines characteristic thermolysis pathways associated with ester bond cleavage and decarboxylation processes. In addition, the relatively flexible polypropylene glycol segments increase the sensitivity of the system to changes in macromolecular architecture.

The investigated system combines thermolabile RAFT fragments, polyester regions with higher thermal stability, and partially cross-linked domains with different local mobility. This results in the formation of overlapping thermal degradation pathways, redistribution of thermolysis stages, and the emergence of architecture-dependent kinetic heterogeneity, which is significantly less pronounced in conventional linear acrylic RAFT systems.

In the present work, the term “macromolecular architecture” is used to describe the structural organization features of the copolymer system, including the ratio of branched and cross-linked fragments, the degree of structural heterogeneity, and the distribution of thermolabile regions formed in the presence of the RAFT agent.

However, despite the large number of studies devoted to RAFT polymerization of vinyl and acrylic systems [1,2,3,4,5,6,10,11,13,14], copolymers based on unsaturated polypropylene glycol maleate polyester resin (p-PGM) and acrylic acid (AA) synthesized under RAFT polymerization conditions remain insufficiently investigated.

Moreover, systematic studies of the thermal degradation of RAFT-modified p-PGM/acrylic acid copolymers using the TG–MS method are currently limited, while the influence of the RAFT agent 2-cyano-2-propyl dodecyl trithiocarbonate (CPDT) on decarboxylation processes and kinetic features of thermolysis has remained largely unexplored.

Thus, the scientific novelty of the present work lies not only in the investigation of RAFT-modified p-PGM:AA copolymers, but also in the comprehensive interpretation of architecture-dependent thermolysis based on a combination of TG–MS analysis, isoconversional kinetics, and DFT modeling.

These copolymers therefore represent a useful model for studying relationships between polymer structure and thermal degradation behavior.

The purpose of the study is to establish the relationship between the concentration of the RAFT agent, the architecture of macromolecules, the morphology and thermal properties of copolymers using ^1^H and ^13^C methods of NMR spectroscopy, TEM and thermogravimetric analysis.

This study demonstrates that branching and partial cross-linking processes occurring under RAFT polymerization conditions lead to redistribution of thermal degradation stages, affect decarboxylation processes, and determine the architecture-dependent kinetic heterogeneity of thermolysis.

## 2. Materials and Methods

### 2.1. Materials

Propylene glycol, maleic anhydride, acrylic acid (AA), benzoyl peroxide (BP), zinc chloride, 1,4-dioxane, and the RAFT agent 2-cyano-2-propyl dodecyl trithiocarbonate (CPDT) were purchased from Sigma-Aldrich (Burlington, MA, USA) and used without further purification.

### 2.2. Synthesis of Polypropylene Glycol Maleate

Polypropylene glycol maleate (p-PGM) was synthesized by polycondensation of maleic anhydride with propylene glycol at 423–453 K according to a previously reported procedure [15]. The molecular weight of the obtained oligomer was determined by gel permeation chromatography (GPC).

### 2.3. Radical Copolymerization of p-PGM with Acrylic Acid

Radical copolymerization of p-PGM with acrylic acid (50:50 mol%) was carried out at 343 K in 1,4-dioxane used as a solvent (1:1 by weight). Benzoyl peroxide (BP) served as the radical initiator. The reaction scheme of the radical copolymerization of p-PGM with acrylic acid and the formation of RAFT-modified copolymers is presented in Scheme S1 in the Supplementary Materials.

Prior to polymerization, the reaction mixture was degassed for 30 min. The ampoules containing the reaction mixture were then sealed and maintained at 343 K for 52 h.

After polymerization, the resulting copolymers were repeatedly washed with dioxane and dried in a vacuum oven at 323–333 K until a constant weight was obtained.

### 2.4. RAFT Polymerization

RAFT polymerization of p-PGM with acrylic acid was carried out under conditions similar to those used for radical copolymerization but in the presence of different concentrations of the RAFT agent CPDT.

The reaction mixtures were purged with an inert gas, sealed in ampoules, and placed in a thermostat maintained at 343 K (±0.10 °C) for 52 h.

After completion of the reaction, the ampoules were cooled and opened. The cross-linked polymers were separated from the mother liquor by filtration and dried in a vacuum oven at 323–333 K until a constant mass was reached.

Branched copolymers were subsequently isolated by precipitation of the mother liquor in alcohol [14].

The chemical structure of the synthesized branched copolymers p-PGM:AA:CPDT and the initial p-PGM oligomer was investigated using ^1^H and ^13^C nuclear magnetic resonance (NMR) spectroscopy on a JNM-ECA JEOL 400 spectrometer (JEOL Ltd., Tokyo, Japan). (399.78 and 100.53 MHz, respectively) with CDCl_3_ as the solvent. Chemical shifts were referenced to the residual solvent signals.

The composition of the synthesized copolymers was determined by high-performance liquid chromatography (HPLC) based on the content of unreacted monomers using a Shimadzu chromatograph (Kyoto, Japan).

Additional structural characterization was performed using infrared (IR) spectroscopy. IR spectra were recorded using KBr pellets prepared by grinding the polymer samples with potassium bromide followed by pressing into tablets. The spectral resolution was 1 cm^−1^, and each spectrum was averaged over 50 scans.

The surface morphology of the synthesized samples was examined using a MIRA3 scanning electron microscope (SEM) (Tescan, Brno, Czech Republic). Transmission electron microscopy (TEM) images were analyzed using Fiji software (ImageJ version 1.53t, National Institutes of Health, Bethesda, MD, USA) after calibration with the scale bar.

The size of the domain structures was evaluated using the equivalent diameter, calculated from the projected area of the domain according to Equation (1):

(1) $$D_{eq}=2\sqrt{\frac{A}{\pi }}$$

where *A* represents the projected area of the domain.

At least 100 domains were analyzed for each sample to obtain statistically reliable size distributions.

Thermal behavior of the synthesized p-PGM:AA copolymers and RAFT-synthesized p-PGM:AA:CPDT copolymers was investigated using a Labsys Evo TG-DTA/DSC thermogravimetric analyzer (SETARAM, France).

Measurements were performed in corundum crucibles within the temperature range of 30–800 °C under an argon atmosphere. The flow rates of the protective and purge gases were 20 and 50 mL min^−1^, respectively.

Thermal analysis experiments were conducted at heating rates of 2.5, 5.0, 7.5, and 10.0 °C·min^−1^. The sample mass was 10.0 ± 0.5 mg. Each experiment was repeated three times (*n* = 3) to ensure reproducibility.

Experimental data were processed and graphical dependences were constructed using OriginPro 9.0 and Python 3.10 (Anaconda) with the NumPy, SciPy, and Matplotlib (v3.10.1) libraries.

Kinetic parameters of thermal decomposition were determined using model-free isoconversional methods [17,18,19,20,21,22,23,24,25,26], including Friedman [24], Ozawa–Flynn–Wall [25,26], and Non-Parametric Kinetics (NPK) [14,18] approaches.

Additionally, model-based kinetic analysis was performed using the Šesták–Berggren equation to evaluate the mechanisms of thermal decomposition.

Statistical significance of the differences between the obtained kinetic parameters was assessed using Student’s *t*-test and one-way analysis of variance (ANOVA) at a significance level of *p* < 0.05 for each degree of conversion α.

### 2.5. Alkaline Hydrolysis of p-PGM:AA:[CPDT] Copolymers

A sample of p-PGM:AA:[CPDT] copolymer (0.5 g) was dissolved in dioxane under stirring until a homogeneous solution was obtained. An aqueous NaOH solution (0.5 M, 20 mL) was then added in excess to provide alkaline hydrolysis conditions. The reaction medium remained alkaline throughout the hydrolysis. The reaction mixture was heated under reflux with continuous stirring for 3 h. After completion of the hydrolysis, the mixture was cooled to room temperature and filtered to separate the insoluble and soluble fractions. The insoluble fraction was washed with distilled water until neutral pH was reached, subsequently washed with ethanol, and dried under vacuum at 50 °C. The soluble fraction was acidified with dilute HCl to pH 2. The resulting hydrolysis products were characterized by ^1^H NMR spectroscopy and gel permeation chromatography (GPC).

### 2.6. Quantum Chemical Calculations

To obtain a molecular understanding of the mechanism of thermal degradation of p-PGM:AA copolymers and the interpretation of TG-MS data, quantum chemical calculations were performed in the framework of the density functional theory (DFT) [27,28,29,30,31,32] using the Gaussian 16 software package [28]. The geometry of the model structures was optimized in two stages. In the first stage, the semi-empirical PM6 method was used for preliminary optimization, followed by a full optimization at the B3LYP/6-311(d,p) level of theory [32]. All optimized structures were confirmed as true minima on the potential energy surface. B3LYP has been extensively validated for systems containing ester, carboxyl, and vinyl functional groups, which are characteristic of the p-PGM:AA copolymers investigated in this work. In addition, previous studies have demonstrated that this level of theory yields consistent trends in HOMO–LUMO gaps and related conceptual DFT descriptors, making it suitable for comparative analysis of structurally similar fragments. The 6-311(d,p) basis set was employed to ensure an adequate description of valence electron distribution and polarization effects, which are essential for capturing bond cleavage processes and electronic redistribution during thermal degradation. Overall, the chosen B3LYP/6-311(d,p) level of theory represents a reasonable and widely accepted choice for mechanistic interpretation and qualitative–semiquantitative analysis of reactivity trends in polymer-related systems.

## 3. Results

Since the copolymerization process simultaneously produces soluble copolymeric macromolecules and an insoluble cross-linked network, complementary characterization techniques were employed to investigate different levels of structural organization. ^1^H NMR spectroscopy, gel permeation chromatography (GPC), and analysis of hydrolysis products were used to characterize the soluble copolymer fraction, whereas swelling measurements and electron microscopy were employed to evaluate the network structure. Thermal analysis was performed for all obtained materials.

### 3.1. Chemical Structure of Branched p-PGM:AA:[CPDT] Copolymers

#### 3.1.1. NMR Characterization of Branched p-PGM:AA:[CPDT] Copolymers

**Scheme 1:**
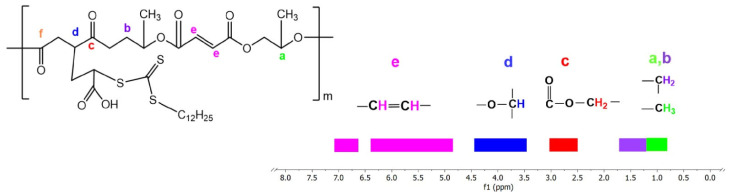
The proposed structure of the p-PGM repeating unit and the branched copolymer, with the designation of proton signals (Ha-He) used in the analysis of ^1^H NMR spectra. The scheme illustrates the possible incorporation of RAFT fragments and the formation of branching regions in the presence of CPDT.

NMR spectroscopy was used to confirm the structure of the synthesized copolymers. The proposed structure of the p-PGM repeating unit with the designation of diagnostic protons (Ha-He) used in the interpretation of ^1^H NMR spectra is shown in Scheme 1.

Scheme 1 presents simplified schematic structures intended to illustrate the possible structural organization of a copolymer system formed in the presence of a RAFT agent. The presented schemes do not reflect all possible microstructural, stereochemical, and topological variants of the obtained copolymers, including possible cis/trans (maleate/fumarate) isomerism, which was not examined in the present study.

**Figure 1:**
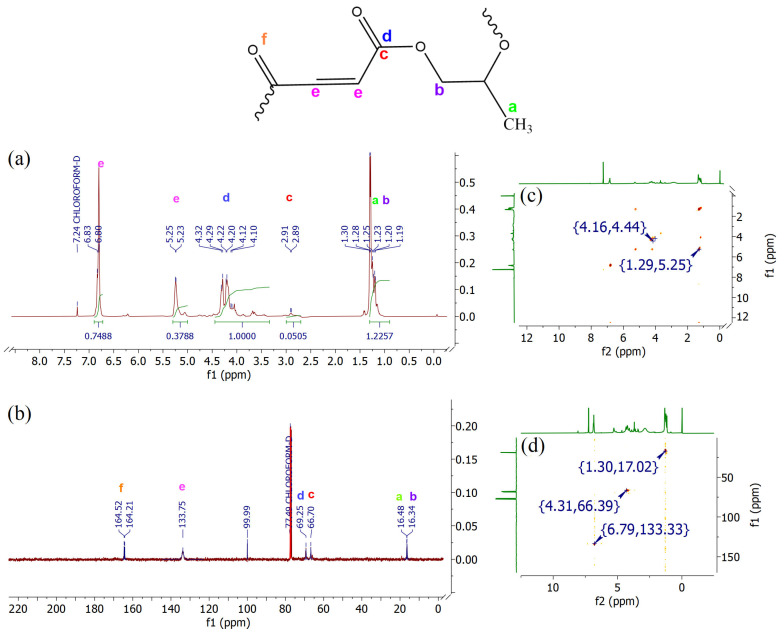
^1^H NMR (**a**), ^13^C NMR (**b**) and two-dimensional ^1^H–^1^H COSY spectra (**c**,**d**) of the p-PGM polyester resin.

The ^1^H NMR spectrum of the starting p-PGM polyester resin (Figure 1a) contains characteristic signals corresponding to the polyester structure and maleate moieties. The multiplet in the region δ = 0.99–1.42 ppm corresponds to the protons of the methyl and methylene groups in the aliphatic moieties of the polypropylene glycol chain (signals a and b). The signals in the region of δ = 2.73–3.01 ppm are assigned to the protons of methylene groups located in the α-position to the carbonyl groups of ester moieties (signal c), while signals at δ = 3.47–4.74 ppm correspond to the protons of methylene (–CH_2_–O–) and methine (–O–CH–) (signal d) groups associated with oxygen atoms in the polyester chain.

In the low-field region, signals are observed at δ = 4.99–6.31 and 6.80–6.83 ppm, corresponding to the vinyl protons (–CH=CH–) of the maleate units (signal e), which indicates the preservation of unsaturated fragments in the structure of the polyester resin.

The ^13^C NMR spectrum (Figure 1b) further confirms the structure of the polyether. The methyl and methylene carbon atoms (signals a and b) are observed at δ = 16.3 ppm. The signals of the carbon atoms of the alkyl moieties linked to the ether groups (signals c and d) are observed in the region of δ ≈ 66.7–69.4 ppm. The vinyl carbon atoms of the maleate fragments (signal e) appear at δ ≈ 133.6 ppm, whereas the signals of the carbonyl carbon atoms of the ester groups are observed at δ ≈ 164.5 ppm (signal f).

The correct assignment of signals in the ^1^H NMR spectrum is confirmed by two-dimensional ^1^H–^1^H COSY spectroscopy data (Figure 1c,d). In the COSY spectrum, cross-peaks are observed between signals in the regions δ = 4.99–6.31 and 6.80–6.83 m (signal e), indicating their scalar interaction. The correlations between signals in regions δ = 3.47–4.74 and 2.73–3.01 ppm confirm that these protons belong to the interconnected methine and methylene groups of the polyester chain. Cross-peaks in the region of δ = 0.99–1.42 ppm (signal a and b) indicate the presence of aliphatic fragments characteristic of propylene glycol links.

**Figure 2:**
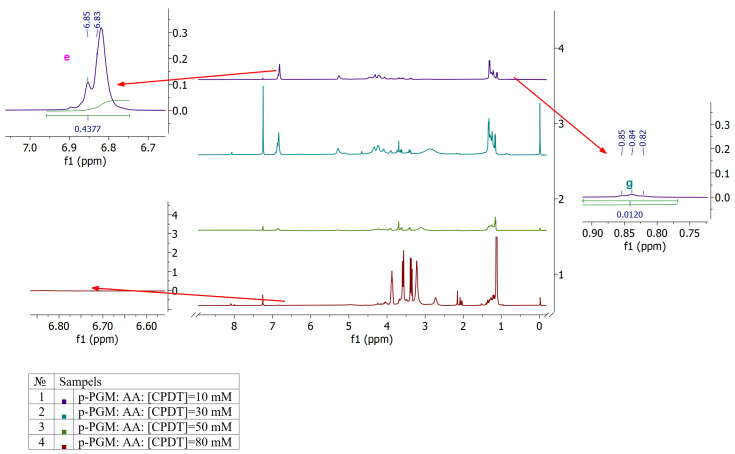
Comparative ^1^H NMR spectra of p-PGM:AA copolymers (49.90:50.10 mol%), synthesized at various concentrations of the RAFT agent CPDT (10–80 mM).

In the ^1^H NMR spectra (Figure 2) of p-PGM:AA copolymers (49.90:50.10 mol%), synthesized at [CPDT] = 10 and 80 mM, differences are observed that reflect the influence of the RAFT agent concentration on the structure of the macromolecules. For the sample synthesized at 10 mM CPDT, weak vinyl proton signals are observed in the region δ = 6.77–6.85 ppm (signal e), indicating incomplete conversion of double bonds. Signals at δ = 5.03–5.21 ppm are also observed and correspond to protons located near double bonds (signal d). Intense signals in the range δ = 4.26–3.65 ppm correspond to –O–CH– and –O–CH_2_– groups of the polyester fragments. In the aliphatic region (δ = 1.27–1.03 ppm), a broad overlapping signal is observed due to –CH_2_– and –CH_3_ protons. The signal at δ = 0.81–0.77 ppm corresponds to terminal methyl groups of the RAFT agent.

For a sample at 80 mM, vinyl proton signals (signal e) are practically absent, which indicates a more complete conversion of double bonds. The main signals are concentrated in the range of δ = 3.88–3.22 ppm, corresponding to the protons of –O–CH– and –O–CH_2_– groups of the polyester moieties. The aliphatic region δ = 1.32–0.82 ppm (signal a and b) is characterized by a pronounced broadening due to the overlap of –CH_2_– and CH_3_ proton signals of the main chain and alkyl fragments of the RAFT agent, while the signal of tertiary methyl groups (signal a) does not appear as a resolved peak. The data obtained show that an increase in the concentration of the RAFT agent is accompanied by an increase in the degree of double-bond conversion and a change in the macromolecular architecture of the copolymers.

The complete ^1^H and ^13^C NMR spectra are presented in the Supplementary Material (Figure S1). The IR and Raman spectra, confirming the presence of ester and unsaturated moieties in the copolymer structure, are presented in the Supplementary Material (Figures S2 and S3).

**Figure 3:**
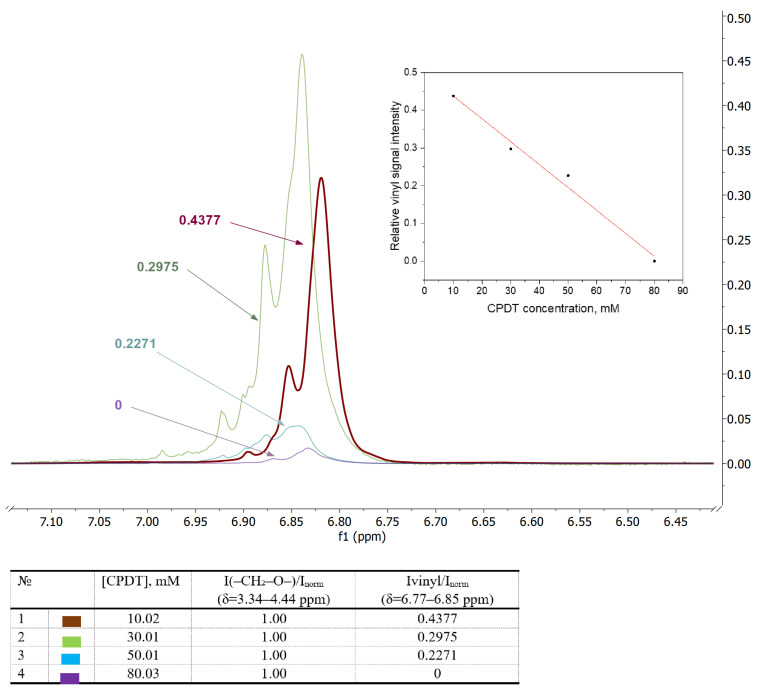
Relative integral intensities of ^1^H NMR signals of p-PGM:AA:[CPDT] copolymers at different CPDT concentrations. Increasing RAFT agent concentration leads to a decrease in vinyl signal intensity.

To further evaluate structural changes in p-PGM:AA:[CPDT] copolymers, relative integral intensities of characteristic ^1^H NMR signals were analyzed. The region of ether methylene protons –CH_2_–O– (δ = 3.34–4.44 ppm) was used as an internal standard and normalized to 1.00. This normalization allowed comparison of relative intensities of vinyl signals at different RAFT concentrations. As shown in Figure 3, increasing CPDT concentration leads to a progressive decrease in the relative intensity of vinyl signals at δ = 6.77–6.85 ppm, indicating a reduction in the content of unreacted double bonds in p-PGM:AA copolymers. These results confirm the influence of CPDT on the incorporation of unsaturated maleate and acrylate fragments into radical copolymerization and are consistent with the formation of a more complex, likely more branched, copolymer architecture.

The ^1^H NMR data indicate changes in the relative abundance of structural fragments with increasing CPDT concentration. However, NMR spectroscopy alone does not provide direct information on the nature of the individual structural elements present in the copolymer architecture. Therefore, alkaline hydrolysis of the polyester segments was carried out, and the resulting products were analyzed to provide additional information on the structure of the copolymer system.

#### 3.1.2. Structural Characterization of Branched p-PGM:AA:[CPDT] Copolymers by Alkaline Hydrolysis

**Figure 4:**
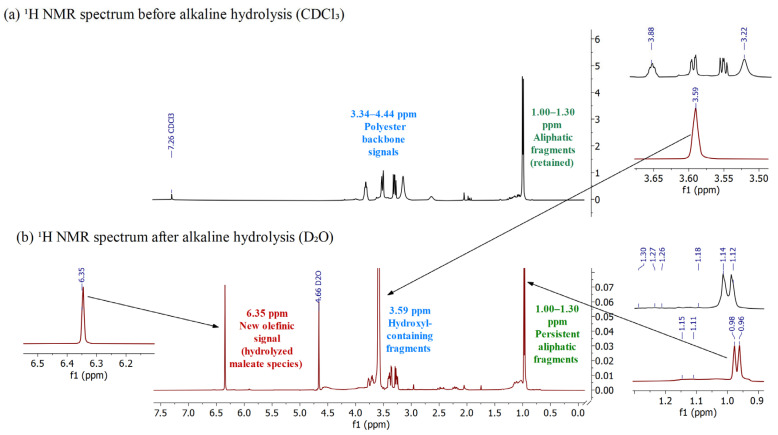
^1^H NMR spectra of p-PGM:AA:[CPDT] = 80 mM before and after alkaline hydrolysis: (**a**) initial copolymer recorded in CDCl_3_; (**b**) hydrolyzed sample recorded in D_2_O. The initial copolymer was analyzed in CDCl_3_, whereas the hydrolyzed sample was recorded in D_2_O due to its increased hydrophilicity following hydrolysis.

To obtain more direct information on the structural fragments comprising the copolymer system, alkaline hydrolysis of the polyester segments was performed. The corresponding ^1^H NMR spectra recorded before and after hydrolysis are presented in Figure 4. Prior to hydrolysis (Figure 4a), the spectrum exhibits characteristic signals of the polyester backbone in the region δ = 3.34–4.44 ppm, together with signals assigned to unsaturated maleate fragments. Following treatment with NaOH (Figure 4b), significant changes are observed in the oxygenated region of the spectrum, indicating cleavage of ester linkages within the p-PGM structure.

After hydrolysis, a new olefinic signal appears at δ = 6.35 ppm, which may be associated with changes in the chemical environment of the maleate units following ester bond cleavage. The persistence of aliphatic signals in the region δ = 1.00–1.30 ppm indicates the presence of structural fragments that remain after hydrolytic degradation of the polyester component.

Thus, alkaline hydrolysis results in cleavage of the ester bonds within the p-PGM polyester backbone, as evidenced by the changes observed in the δ = 3.34–4.44 ppm region and the appearance of a new olefinic signal at δ = 6.35 ppm. The retention of a portion of the aliphatic signals after hydrolysis suggests the presence of structural fragments resistant to hydrolytic degradation. These results confirm the effective hydrolytic cleavage of the polyester component and provide additional information on the structural features of the p-PGM:AA:[CPDT] copolymers.

**Figure 5:**
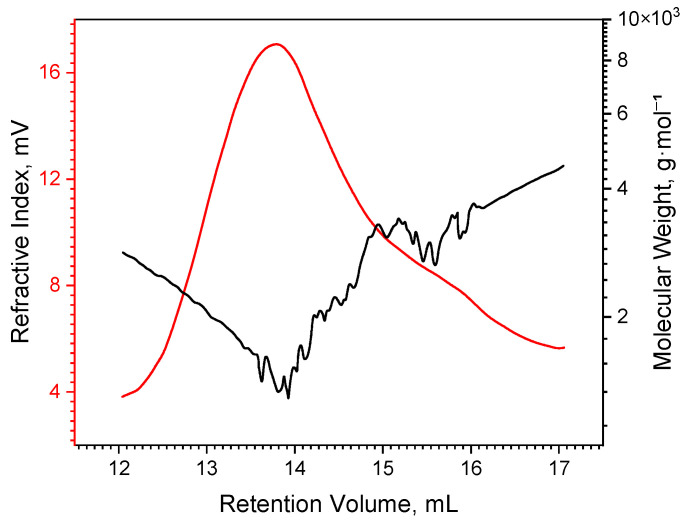
GPC chromatogram of the hydrolysis products obtained from the p-PGM:AA:[CPDT] copolymer (CPDT = 80 mM) and the corresponding molecular weight distribution.

To further characterize the hydrolysis products, gel permeation chromatography (GPC) analysis was performed (Figure 5). The hydrolyzed sample exhibited a number-average molecular weight (*M_n_*) of 1370 g·mol^−1^ and a weight-average molecular weight (*M_w_*) of 1417 g·mol^−1^, corresponding to a dispersity of *Đ* = 1.035. The low dispersity value indicates a relatively narrow molecular weight distribution of the species present after hydrolysis. The observed molecular weight values are consistent with cleavage of the ester linkages within the polyester component of the copolymer under alkaline hydrolysis conditions. These results are consistent with the ^1^H NMR data, which suggest changes in the oxygen-containing structural fragments following alkaline hydrolysis.

Although alkaline hydrolysis provides information on changes in the polyester component of the copolymer, it does not directly reveal variations in the distribution of structural fragments among the samples. Therefore, the effect of CPDT concentration on copolymer composition was further examined using normalized ^1^H NMR signal integrals.

#### 3.1.3. Semi-Quantitative Analysis of Structural Changes in Branched p-PGM:AA:[CPDT] Copolymers by ^1^H NMR Spectroscopy

The broadband proton-decoupled ^13^C NMR spectra obtained in this study do not permit quantitative differentiation of linear, terminal, and branched structural units according to the classical Frey method for determining the degree of branching (DB) [33]. Consequently, direct determination of DB for the investigated copolymers was not possible. Instead, structural variations among the samples were assessed semi-quantitatively using normalized integrated intensities of selected ^1^H NMR signals [34,35,36,37,38,39].

To characterize relative structural changes, two parameters were defined:

$${BI}^{*}=\frac{I_{0.90-1.30}}{I_{3.34-4.44}}$$

and

$${RI}^{*}=\frac{I_{0.92-0.80}}{I_{3.34-4.44}}$$

where the δ = 3.34–4.44 ppm region corresponds to signals arising from the polyester backbone, while the δ = 0.90–1.30 and 0.80–0.92 ppm regions contain signals associated with methyl-containing structural fragments. It should be noted that the BI* and RI* parameters do not represent quantitative measures of the degree of branching. Unlike classical hyperbranched systems, in which dendritic, linear, and terminal units can be individually identified and used for calculation of the degree of branching (*DB*) [37], the copolymers investigated in the present study exhibit substantial overlap of signals originating from different structural fragments. Therefore, these parameters should be regarded solely as relative descriptors of structural variation. The BI* and RI* values calculated from the ^1^H NMR data are summarized in Table 1.

At the same time, the relative intensity of the vinyl proton signals attributed to maleinate fragments in the region δ = 6.77–6.85 ppm decreased with increasing CPDT concentration, consistent with progressive consumption of the unsaturated fragments during copolymerization. The BI* parameter changes with CPDT concentration and reflects variations in the relative contribution of methyl-containing structural fragments. Because the δ = 0.90–1.30 ppm region includes overlapping signals from multiple structural environments, including aliphatic polyester segments and RAFT-derived structural elements, a strictly monotonic relationship between BI* and CPDT concentration would not be expected. In contrast to BI*, the RI* parameter generally increases with increasing CPDT concentration. Because the integrated intensity of NMR signals is proportional to the number of contributing protons, this trend is consistent with an increased relative contribution of terminal methyl-containing fragments compared with the polyester backbone. Thus, the ^1^H NMR data indicate systematic changes in the relative abundance of structural fragments in the copolymers with varying CPDT concentration. Although direct determination of the degree of branching according to the Frey approach is not possible, the results suggest changes in copolymer structure as a function of the RAFT polymerization conditions. To evaluate the molecular weight characteristics of the soluble copolymer fraction, gel permeation chromatography (GPC) analysis was subsequently performed.

#### 3.1.4. Molecular Weight Characteristics of Branched p-PGM:AA:[CPDT] Copolymers

**Figure 6:**
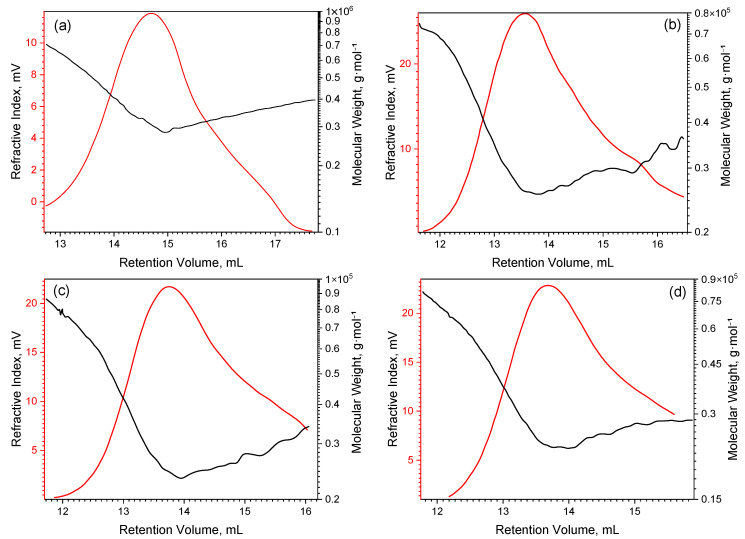
GPC chromatograms of p-PGM:AA:[CPDT] copolymers synthesized at different CPDT concentrations (10–80 mM): (**a**) 10 mM; (**b**) 30 mM; (**c**) 50 mM; (**d**) 80 mM. Increasing RAFT agent concentration leads to decreased molecular weight and narrowing of molecular weight distribution.

As shown in Figure 6 and Table 2, the molecular weight characteristics of the p-PGM:AA:[CPDT] copolymers depend on the CPDT concentration. For the initial polyester resin p-PGM, the weight-average molecular weight (*M_w_*) was 2.44 × 10^3^ g·mol^−1^ [8,9]. As evident from Figure 6, increasing the CPDT concentration results in a progressive shift in the GPC chromatograms toward higher elution volumes, consistent with a decrease in the molecular weight of the analyzed samples. At the same time, the relatively low dispersity values (*Đ* = 1.152–1.139) indicate comparatively narrow molecular weight distributions.

The dependence of the molecular weight characteristics on conversion was examined only for one RAFT agent concentration (CPDT = 80 mM) (Figure 6). As shown in Figure 6, the number-average molecular weight (*M_n_*) increases approximately linearly with increasing monomer conversion, while the dispersity (*M_w_*/*M_n_*) decreases and approaches a value of approximately 1.14. This behavior is consistent with chain-transfer processes occurring under RAFT copolymerization conditions and the formation of copolymer fractions with a more uniform molecular weight distribution.

The observed decrease in *M_w_* is consistent with an increased contribution of chain-transfer processes during RAFT copolymerization. These results are in agreement with the ^1^H NMR spectroscopic data.

**Figure 7:**
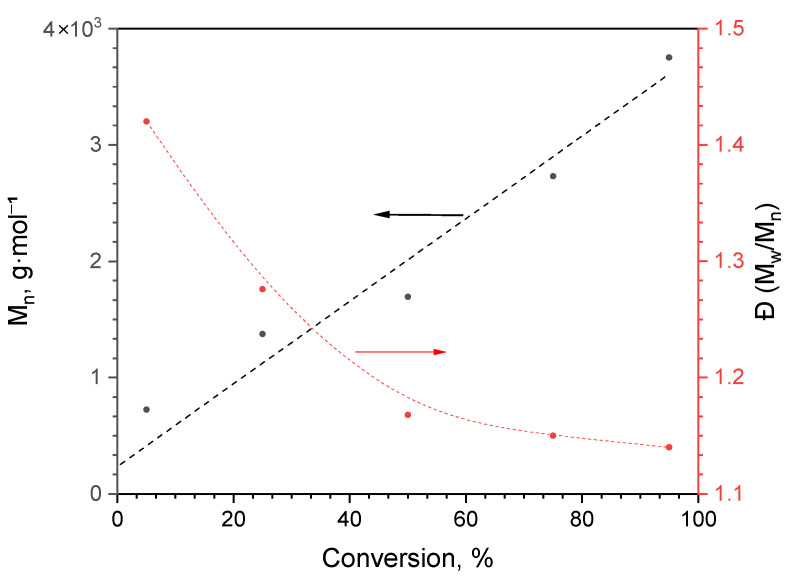
Molecular weight characteristics of p-PGM:AA copolymers synthesized in the presence of CPDT (80 mM). The plot shows the experimental values of the number-average molecular weight (*M_n_*, left axis) and dispersity (*Đ* = *M_w_*/*M_n_*, right axis). The lines are intended to guide the eye and illustrate the overall trends in the measured parameters.

The conversion–molecular weight relationship was examined for a single RAFT agent concentration ([CPDT] = 80 mM) (Figure 7). The number-average molecular weight increased with conversion, whereas the dispersity decreased progressively. This behavior is consistent with that expected for a RAFT-mediated polymerization process.

It should be noted that GPC and NMR analyses provide information primarily on the soluble fraction of the copolymers. Additional characterization of the insoluble network component was performed using swelling experiments and electron microscopy.

### 3.2. Characterization of the Network Structure of Cross-Linked p-PGM:AA:[CPDT] Copolymers

**Figure 8:**
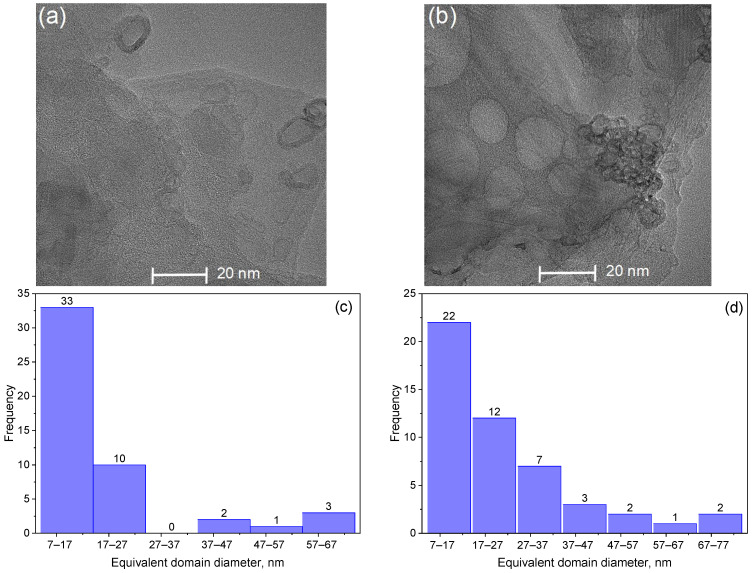
TEM images of p-PGM:AA copolymers: (**a**) sample synthesized without CPDT; (**b**) sample synthesized at [CPDT] = 80 mM; (**c**,**d**) equivalent domain diameter distributions. The sample prepared in the presence of CPDT shows a broader domain size distribution and a greater contribution from larger domains.

The morphology of the cross-linked copolymers was investigated by transmission electron microscopy (TEM). The analysis was performed for samples synthesized in the absence and in the presence of CPDT. For the p-PGM:AA sample prepared without CPDT (Figure 8a,c), domains with equivalent diameters predominantly in the range of 7–17 nm were observed. According to the size distribution, the majority of domains fall within this range, whereas the contribution of larger structures is relatively small. For the p-PGM:AA:[CPDT] sample ([CPDT] = 80 mM) (Figure 8b,d), a broader domain size distribution was observed, with an increased proportion of structures in the 17–37 nm range. In addition, domains with equivalent diameters of up to 67–77 nm were detected. The average equivalent domain diameter increased from 18.6 ± 13.6 nm for p-PGM:AA to 24.2 ± 15.7 nm for p-PGM:AA:[CPDT] ([CPDT] = 80 mM). The observed differences in domain size distribution are consistent with changes in the morphology of the copolymer system in the presence of CPDT.

**Figure 9:**
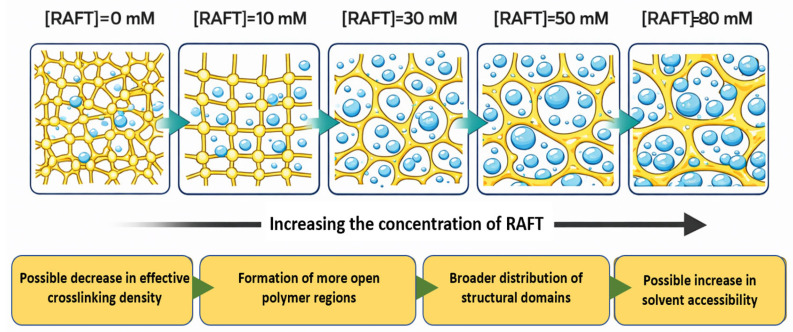
Qualitative schematic illustration of possible structural differences between p-PGM:AA copolymers synthesized in the absence and presence of CPDT and their corresponding swelling behavior. The scheme is intended for illustrative purposes only and does not represent a quantitative structural model.

For qualitative comparison of the observed morphology with macroscopic swelling behavior, previously reported swelling degree values are summarized in Table 3 [9]. As shown in Table 3 and the size distribution histograms (Figure 8c,d), the sample synthesized in the presence of the RAFT agent exhibits a broader domain size distribution and the appearance of larger structural entities. At the same time, the increased swelling degree may reflect a change in the structural organization of the system and enhanced accessibility of the polymer network to solvent molecules. The corresponding trends are schematically illustrated in Figure 9, which is qualitative in nature and depicts the possible influence of RAFT agent concentration on copolymer structure formation.

Differences in the molecular weight characteristics and morphology of the copolymers suggest corresponding differences in their thermal behavior. Accordingly, the branched and cross-linked copolymers were further characterized by TG/DTG analysis.

### 3.3. Thermal Behavior of Branched and Cross-Linked p-PGM:AA:[CPDT] Copolymers

### 3.3.1. Behavior of Copolymers in Thermogravimetric Analysis (TG/DTG)

**Figure 10:**
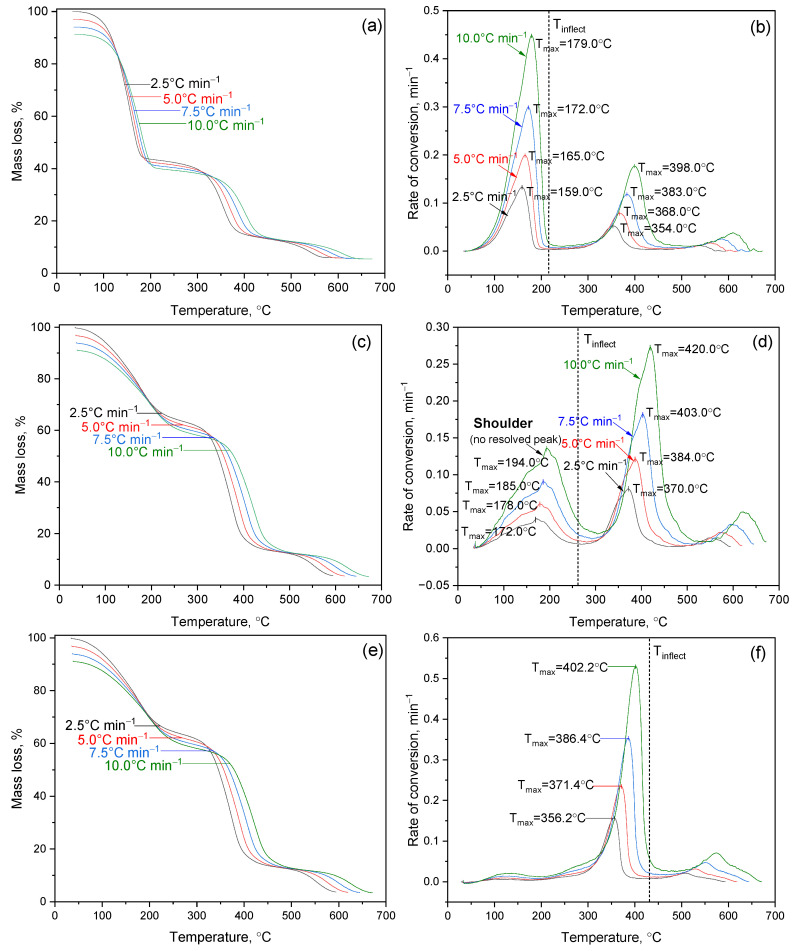

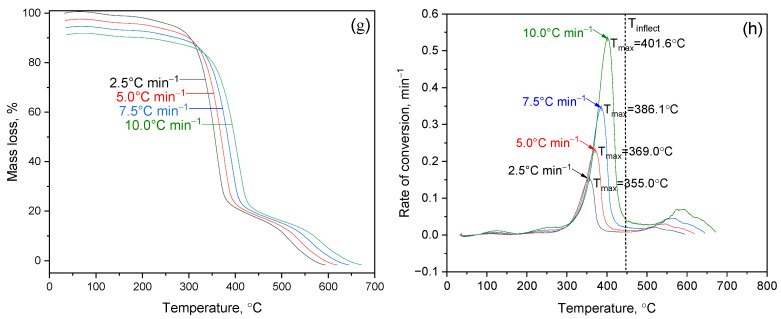
Thermogravimetric (TG; (**a**,**c**,**e**,**g**)) and differential thermogravimetric (DTG; (**b**,**d**,**f**,**h**)) curves of branched and cross-linked p-PGM:AA copolymers recorded at various heating rates (*β* = 2.5–10.0 °C·min^−1^): (**a**,**b**) branched p-PGM:AA copolymer:[CPDT] = 10 mM; (**c**,**d**) branched p-PGM:AA copolymer:[CPDT] = 80 mM; (**e**,**f**) cross-linked p-PGM:AA copolymer (without CPDT); (**g**,**h**) cross-linked p-PGM:AA copolymer:[CPDT] = 80 mM.

Figure 10 shows the TG and DTG curves of branched and cross-linked copolymers of p-PGM:AA synthesized without CPDT and in the presence of RAFT agent at concentrations of 10 and 80 mM. The measurements were carried out at heating rates of 2.5–10.0 °C·min^−1^. The TG curves (Figure 10a,c,e,g) reflect the change in the relative mass of the samples during heating, while the DTG curves (Figure 10b,d,f,h) characterize the rate of mass loss and allow the identification of individual stages of thermal de-structuring.

Branched copolymers of p-PGM:AA:[CPDT] show multi-stage degradation (Figure 10a–d).

In the range of ~160–190 °C, a low-temperature stage is recorded, caused by the decomposition of thermolabile side chains and the initial fragmentation of polyester bonds of the polyester chain. The main stage of degradation occurs in the range of ~350–420 °C and is associated with the destruction of the polyester backbone of the copolymer. As the heating rate increases, the DTG peaks shift to higher temperatures (Figure 10b,d), indicating that the process is kinetically controlled.

An increase in the concentration of RAFT agent from 10 to 80 mM is accompanied by a change in the parameters of the main decomposition stage: the amplitude of the DTG peak increases and its temperature width decreases (Figure 10b,d), which indicates the localization of the temperature interval of chain decay. The observed changes are associated with the transformation of the macromolecular architecture and the redistribution of thermolabile fragments in the copolymer structure.

The p-PGM:AA cross-linked copolymer obtained without the RAFT agent (Figure 10e,f) exhibits the main degradation step in a comparable temperature range (~356–402 °C), but has a significantly lower temperature width of the DTG peak compared to branched systems. This reflects the narrower temperature range of chain decay and the pronounced cooperative nature of destruction under conditions of a rigidly fixed cross-linked structure.

The p-PGM:AA:[CPDT] = 80 mM cross-linked copolymer (Figure 10g,h) is characterized by an increase in the amplitude of the DTG peak and a rise in the contribution of the main stage to the total mass loss, indicating the dominant role of chain breakdown in the formation of the thermal degradation profile of this system.

The inclusion of thermolabile moieties such as thiocarbonylthio groups (–S–C(=S)–S–) and carboxylic groups of acrylic acid in the copolymer structure causes an additional low-temperature decomposition step. Their destruction is accompanied by the formation of active radical centers that initiate further breakdown of the macromolecular chain. At the same time, decarboxylation of carboxyl groups leads to the isolation of CO_2_, which is consistent with the TG-MS analysis.

The quantitative thermal degradation parameters of the copolymers tested are shown in Table 4.

From an applied perspective, thermogravimetric parameters enable the determination of acceptable temperature–time profiles for the processing and use of p-PGM:AA copolymers. The higher values of |dm/dT|ₘₐₓ for cross-linked systems (1.32–1.37%·°C^−1^) compared to branched systems (0.45–0.73%·°C^−1^) reflect a more intense course of the main degradation stage at temperatures close to T_max_, which requires more precise control of the temperature regime in the region of the main degradation.

The presence of a low-temperature stage in the range of 150–200 °C for RAFT-modified branched copolymers indicates the possibility of partial structural degradation at temperatures significantly below the T_max_ of the main stage. In this regard, during drying and heat treatment, it is necessary to limit the holding time in this temperature range.

The parameters T_max_, ΔT_1_/_2_, |dm/dT|ₘₐₓ and the contribution of the main stage can be used as quantitative criteria for assessing the thermal stability of copolymers and selecting optimal processing conditions. Based on these parameters, a safe processing temperature window is determined, within which the structural stability of the materials is maintained.

Thermogravimetric analysis made it possible to establish temperature ranges and basic parameters for the thermal decomposition of copolymers. The composition of the volatile products formed by heating was further analyzed by thermal programmed mass spectrometry (TP-MS).

Branched and cross-linked copolymers based on polypropylene glycol maleate (p-PGM) and acrylic acid (AA), obtained both in the presence of the RAFT agent CPDT (10 and 80 mM) and without it, served as research objects. It has been shown that the thermal decomposition of copolymers proceeds through a multi-stage mechanism in the range of 50–600 °C with the formation of characteristic volatile products identified by *m*/*z* values.

**Figure 11:**
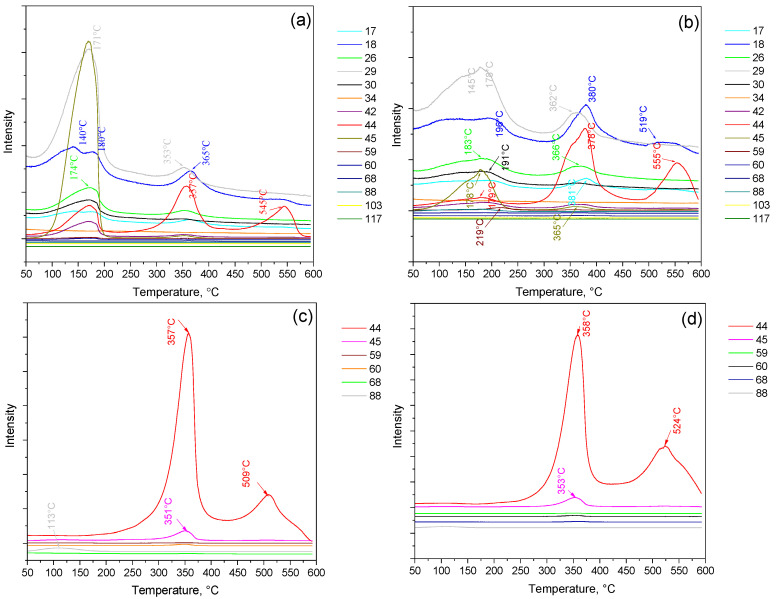
TG–MS profiles of gaseous products released during the thermal decomposition of p-PGM:AA copolymers: (**a**) branched p-PGM:AA:[CPDT] (10 mM); (**b**) branched p-PGM:AA:[CPDT] (80 mM); (**c**) cross-linked p-PGM:AA (without CPDT); (**d**) cross-linked p-PGM:AA:[CPDT] (80 mM). For RAFT-modified samples, an additional low-temperature evolution of CO_2_ and oxygen-containing fragments is observed.

In the range of 50–250 °C (Figure 11a), signals are observed corresponding to the release of water (*m*/*z* = 18), hydrocarbon and vinyl fragments (*m*/*z* = 26–30), as well as oxygen-containing products of decarboxylation and the decomposition of acetate and maleate groups (*m*/*z* = 42–45). The predominance of the CO_2_ signal (*m*/*z* = 44) indicates the onset of decarboxylation of carboxyl and ester fragments of the polymer structure.

In the temperature range of 250–400 °C, for the p-PGM:AA:[CPDT] = 10 mM copolymer, an increase in the CO_2_ (*m*/*z* = 44) signal is observed, attributed to the degradation of the acrylate and maleate units. At the same time, the signals for water (*m*/*z* = 18) and hydrocarbon fragments (*m*/*z* = 26–29) are retained, reflecting the further degradation of the side chains and the initial fragmentation of the main macromolecular chain. At the final stage (400–600 °C), the composition of volatile products is almost entirely determined by the evolution of CO_2_ (*m*/*z* = 44), which corresponds to the deep destruction of oxygen-containing fragments and the completion of the high-temperature thermal decomposition step.

With a high concentration of RAFT agent (80 mM, Figure 11b), the content of vinyl residues and defective fragments decreases, which is accompanied by a decrease in the intensity of the early stage of thermal destruction (100–200 °C). The main decomposition step in the range of 300–400 °C is due to the breaking of ester bonds (–C(=O)–O–), accompanied by decarboxylation and isolation of CO_2_.

In the range 100–200 °C, a decrease in the intensity of the *m*/*z* = 26–30 (C_2_H_2_^+^, C_2_H_3_^+^, C_2_H_4_^+^, C_2_H_5_^+^) signals, corresponding to hydrocarbon and vinyl fragments, is also observed as the concentration of the RAFT agent increases. The combination of these changes reflects the effect of RAFT modification on the mechanism of thermal degradation of copolymers.

Compared with RAFT-modified systems, the p-PGM:AA cross-linked copolymer prepared without CPDT (Figure 11c) exhibits a simpler thermal decomposition profile. Gas emission is limited to two temperature intervals (250–450 °C and 450–600 °C), with CO_2_ (*m*/*z* = 44) remaining the main degradation product. The limited number of temperature zones and the low intensity of the accompanying signals reflect a more homogeneous nature of the thermal decomposition and the predominant breakdown of the main polymer chain.

The cross-linked copolymer p-PGM: AA:[CPDT] = 80 mM (Figure 11d) also exhibits a two-stage degradation profile, but is characterized by an increased CO_2_ (*m*/*z* = 44) intensity and an additional contribution of acetate fragments (*m*/*z* = 45) in the middle temperature range. The totality of the observed changes indicates the effect of RAFT modification on the mechanism of thermal destruction and an increase in the yield of volatile products while maintaining a dense cross-linked architecture.

The use of the CPDT RAFT agent in the synthesis of p-PGM:AA copolymers leads to changes in their molecular architecture and thermal behavior profile. The formation of branched macromolecules containing side-chain and terminal thiocarbonylthio fragments (–S–C(=S)–S–) is accompanied by a redistribution of the temperature ranges of the decomposition stages. For RAFT-modified samples, the yield of CO_2_ and acetate fragments is observed as early as the 150–200 °C range, due to the degradation of thermolabile functional groups, whereas the temperature at which the main stage of polyester backbone degradation occurs remains virtually unchanged.

As the CPDT concentration is increased to 80 mM, a redistribution of the contributions of the individual decomposition stages occurs, accompanied by the appearance of a signal at *m*/*z* = 45 and an increase in CO_2_ yield in the medium- and high-temperature ranges. In contrast, cross-linked copolymers synthesized without CPDT exhibit a narrower spectrum of volatile products and a predominantly two-stage degradation mechanism with dominant CO_2_ evolution in the 250–450 and 450–600 °C ranges.

The RAFT modification thus alters the thermal degradation profile and redistributes the energy contributions of the individual stages of the multi-stage process without significantly shifting the main stage of macromolecular backbone degradation. Based on TG–MS data, probable pathways for the thermal decomposition of copolymers have been proposed, involving sequential processes of dehydration, decarboxylation, fragmentation of ether bonds, and the destruction of fragments containing RAFT agent residues, over a wide temperature range of 50–600 °C.

**Scheme 2:**
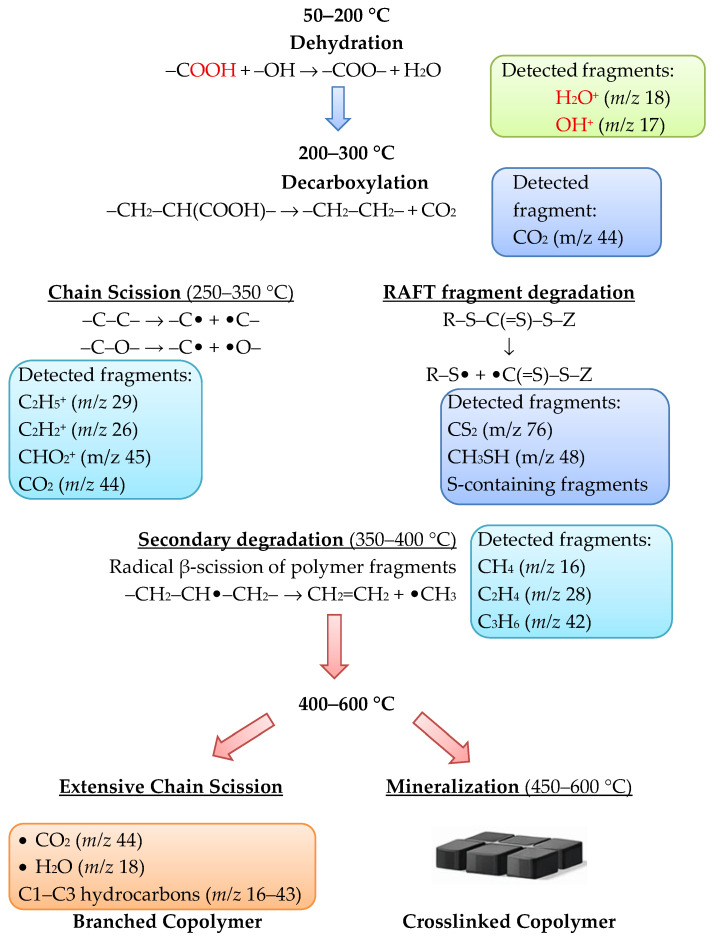
Schematic representation of the multi-stage thermal degradation mechanism of p-PGM:AA:[CPDT] copolymers in the range of 50–600 °C by TG-MS analysis.

The proposed multi-stage degradation pathway derived from the TG–MS analysis is schematically illustrated in Scheme 2.

Although TG–MS analysis enables the identification of the principal volatile products formed during thermal degradation, this technique does not provide direct insight into the possible bond cleavage pathways or the relative energetic characteristics of individual decomposition stages. Therefore, density functional theory (DFT) calculations were additionally performed to qualitatively evaluate the decarboxylation processes.

### 3.3.2. Quantum-Chemical Analysis of Thermal Decomposition Pathways

To assess the possible influence of RAFT-derived fragments on thermal degradation, DFT calculations were carried out on model structures containing a maleinate unit with and without a RAFT fragment. The models represent simplified fragments of the copolymer system and are intended solely to provide qualitative information on possible degradation pathways.

The calculated electronic parameters are listed in Table 5. Incorporation of the RAFT fragment is associated with a reduction in the HOMO–LUMO energy gap and a decrease in global hardness, together with an increase in global softness, indicating changes in the electronic structure of the model systems.

**Figure 12:**
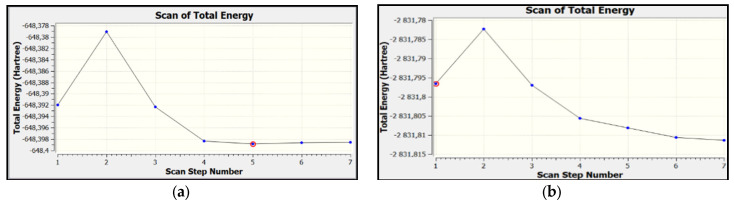
Energy profiles along the C16–C19 bond dissociation coordinate illustrating the effect of the RAFT fragment: (**a**) maleate fragment; (**b**) maleate fragment containing the RAFT fragment.

**Figure 13:**
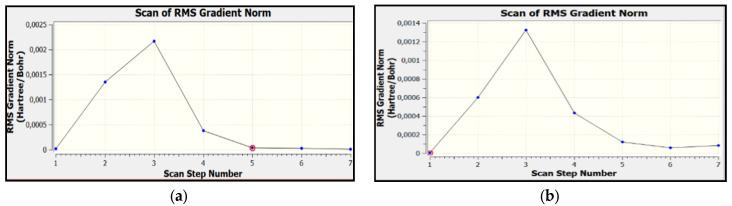
Gradient norm change (RMS) during C–C bond breaking (C16–C19): (**a**) maleate fragment; (**b**) maleate fragment in the presence of the RAFT fragment. The obtained profiles suggest that the presence of the RAFT fragment may influence the bond dissociation behavior.

To examine possible bond cleavage pathways during thermal degradation, the potential energy surface was scanned along the C16–C19 bond dissociation coordinate. The calculated energy profiles (Figure 12) suggest the presence of an activation barrier associated with the investigated bond dissociation process. The corresponding changes in the gradient norm (Figure 13) are consistent with the presence of a high-energy region along the investigated bond dissociation coordinate.

The calculations suggest that the presence of the RAFT fragment may influence the energetic characteristics of the investigated bond dissociation process. These differences are consistent with the changes in thermal behavior observed by TG/DTG analysis.

Overall, the quantum-chemical results complement the experimental thermal analysis data and provide qualitative support for the interpretation of the thermal degradation behavior of the copolymers. The calculations are intended solely to illustrate possible trends in the energetic characteristics of model reactions and should not be regarded as direct proof of the thermal degradation mechanism.

### 3.3.3. Isoconversional and Kinetic Analysis of Thermal Decomposition

For a more detailed assessment of the kinetic features of thermolysis and for comparison of the experimental energetic parameters with the results of DFT modeling, an isoconversional analysis of the TG curves was performed using the Friedman [24] and Ozawa–Flynn–Wall [25,26] methods. As a result, the effective activation energy at different conversion degrees was determined and the values of *E_a_*(*α*) were compared with the calculated decarboxylation barrier.

**Figure 14:**
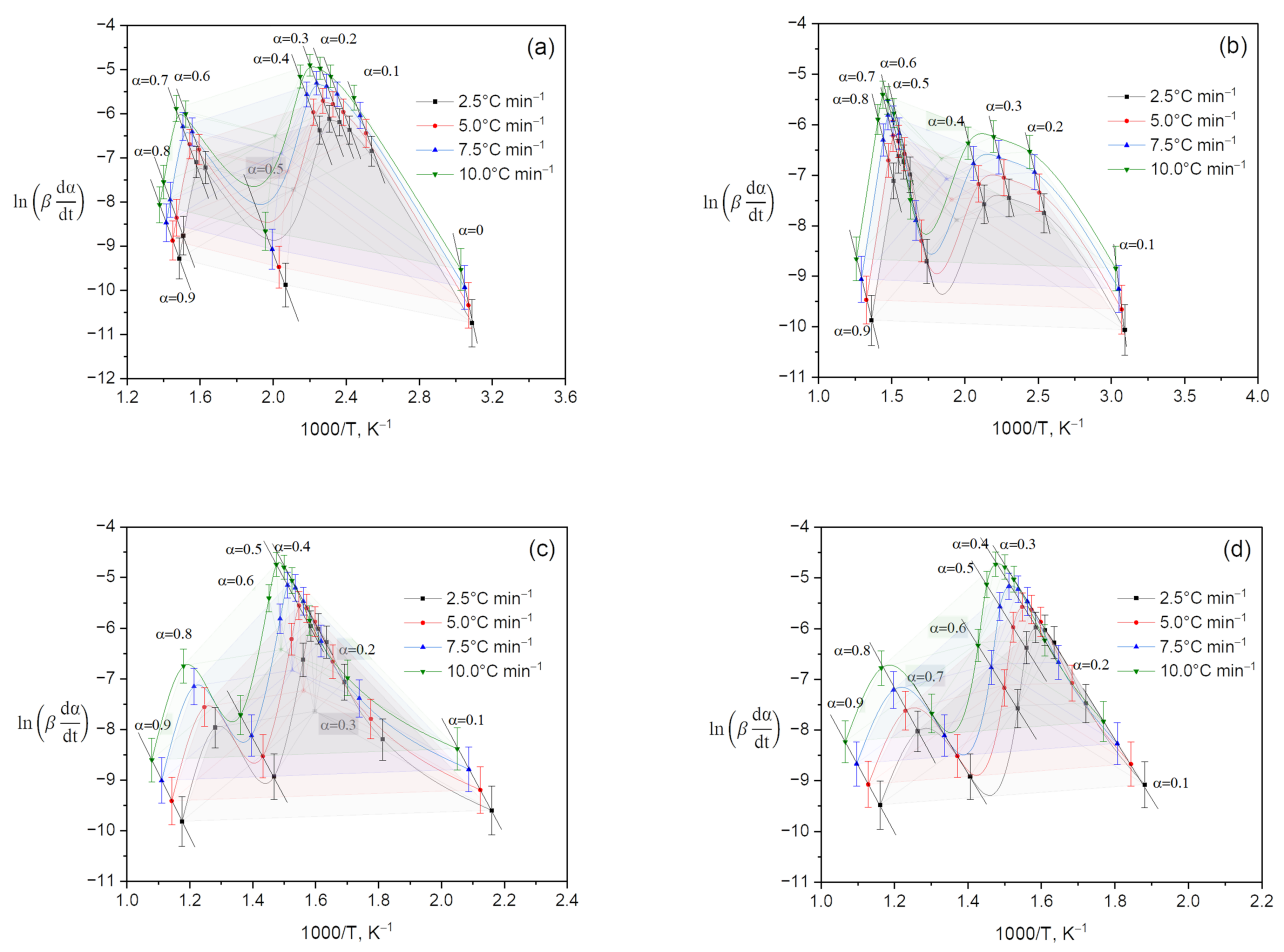
Friedman relationships of ln(*β·dα/dT*) to 1000/T: (**a**) branched p-PGM:AA: [CPDT] (10 mM); (**b**) branched p-PGM:AA: [CPDT] (80 mM); (**c**) cross-linked p-PGM:AA (without CPDT); (**d**) cross-linked p-PGM:AA: [CPDT] (80 mM).

Figure 14 shows the dependence of ln(*β* d*α*/d*T*) on 1000/*T*, plotted using the Friedman method for α = 0.1–0.9 conversions at heating rates of 2.5–10 °C·min^−1^. For each fixed degree of transformation, the dependencies are linear, which confirms the applicability of the isoconversional approach. The activation energy is determined by the slope of the lines (−*E_a_/R*).

The obtained *E_a_*(*α*) values (Table 6) change systematically as the degree of conversion increases. Since α increases monotonically with temperature during linear heating, the dependence of *E_a_*(*α*) reflects a sequential change in reaction stages. The decrease in *E_a_* in the region of low α is associated with the involvement of less thermally stable structural fragments in the initial stage of decomposition, whereas the subsequent increase in *E_a_* is due to the involvement of more stable elements of the polymer matrix.

The TG–MS results, isoconversional analysis, and DFT calculations indicate the multi-stage nature of the thermolysis of p-PGM:AA copolymers. The obtained data also show that the presence of the RAFT agent may influence the distribution of individual stages of thermal degradation. Systems synthesized in the presence of the RAFT agent are characterized by thermolabile thiocarbonylthio and maleate fragments, which is manifested in an earlier release of CO_2_ and acetate products detected by TG–MS.

The corresponding decrease in activation energy in the region of low conversion α is consistent with the calculated decarboxylation barrier, whereas the increase in *E_a_*(*α*) at later stages reflects the involvement of more thermally stable elements of the polyester backbone.

The combination of experimental TG–MS data, isoconversional kinetic analysis, and quantum-chemical modeling enables a coherent description of the thermal decomposition processes and allows evaluation of the influence of macromolecular organization formed in the presence of the RAFT agent on the kinetic profile of thermolysis.

The (*E_a_*) activation energy values calculated by Friedman (FR) and Ozawa–Flynn–Wall (OFW) isoconversional methods at *α* = 0.1–0.9 are given in Table 6. For the branched copolymer (Figure 14a, 10 mM), *E_a_* (FR) decreases from 207.6 to 184.1 kJ·mol^−1^ with increasing α up to 0.7, followed by a plateau, reflecting a change in the kinetic behavior of the system during thermolysis.

For the (Figure 14b, 80 mM) sample, the initial stage is characterized by a significantly higher barrier (*E_a_* = 329.2 kJ·mol^−1^ by FR and 369.7 kJ·mol^−1^ by OFW at α = 0.1), after which the *E_a_* values decrease and stabilize in the range of ~185–190 kJ·mol^−1^. This profile is consistent with a multi-stage degradation mechanism and possible overlap of reaction steps at the early stages of decomposition.

The cross-linked copolymer (Figure 14c) exhibits a more uniform distribution of *E_a_* (180–202 kJ·mol^−1^ by FR) over the entire α range, indicating a more homogeneous thermal degradation behavior associated with a more uniform distribution of cross-linked fragments within the polymer structure. The increase in *E_a_* at α ≈ 0.9 is associated with the destruction of the most thermostable elements of the cross-linked structure. For the (Figure 14d, 80 mM) sample, the increase in *E_a_* at α ≥ 0.7 indicates the involvement of more stable structural elements in the later stages of thermal decomposition.

Overall, the *E_a_*(α) dependence is characterized by a decrease at the initial stages followed by stabilization at higher conversion degrees, reflecting changes in the dominant thermolysis pathways.

The change range of *E_a_*(*α*) is most pronounced for branched copolymer at 80 mM (Δ*E_a_* ≈ 143.6 kJ·mol^−1^), while for cross-linked copolymer it is much smaller (≈21 kJ·mol^−1^), which indicates a more uniform kinetic profile.

An increase in RAFT agent concentration is accompanied by an average increase in *E_a_* of approximately 7.8% for branched systems and only ~2.7% for cross-linked systems. The OFW method systematically yields higher *E_a_* values (by 10–21%) compared to the Friedman method. Low standard deviation values (*SD* ≤ 1.3 kJ·mol^−1^; *CV* < 1%) confirm the reproducibility of the obtained kinetic parameters.

From an applied perspective, the identified *E_a_*(*α*) relationships and the parameters of the kinetic model enable the prediction of the thermal stability of copolymers under various heating conditions. The wide range of *E_a_* values for branched systems synthesized in the presence of the RAFT agent (up to 143.6 kJ·mol^−1^ according to the Friedman method) reflects their kinetic heterogeneity. This also indicates an increased sensitivity of such systems to temperature variations at the initial stages of thermolysis.

For cross-linked copolymers, a significantly narrower *E_a_* range (≈21 kJ·mol^−1^) is observed, which indicates a more predictable decomposition behavior.

The *E_a_*, ln*A*, *m* and *n* parameters can be used as a quantitative basis for assessing permissible temperature–time processing conditions and predicting the thermal stability of materials.

The kinetic interpretation is based on the combined analysis of TG data using the isoconversional methods of Friedman [24] and Ozawa–Flynn–Wall [25,26], non-linear model analysis and singular value decomposition (SVD) [14,18]. This approach enables the analysis of the *E_a_*(α) dependence without prior specification of a kinetic model. In addition, it allows estimation of the number of dominant kinetic components and optimization of model parameters for describing overlapping stages of thermolysis.

**Scheme 3:**
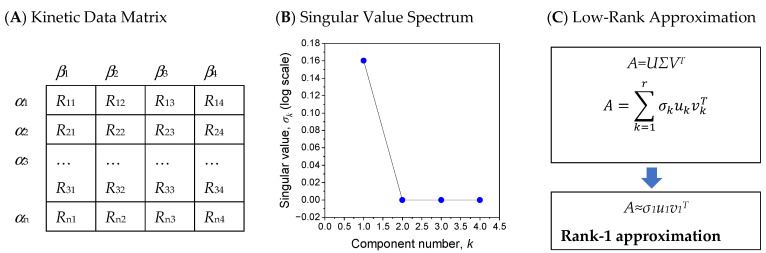
SVD analysis of the kinetic data matrix. (**A**) Construction of the matrix $R(\alpha_{i},\beta_{j})$. (**B**) Singular value spectrum (log scale). (**C**) Low-rank approximation (rank-1) of the kinetic matrix.

The sequence of TG data processing and the kinetic analysis methods used are shown in Scheme 3.

The TG curves obtained at various heating rates were converted into conversion degree dependences *α*(*T*), after which the reaction rate *R*(*α*,*T*) = d*α*/d*t* was calculated. Interpolation at fixed *α* values for each *β* heating rate was used to form the kinetic matrix *Aᵢⱼ* = *R*(*αᵢ*, *βⱼ*), representing the experimental data as a discretized surface *R*(*α*,*T*).

**Figure 15:**
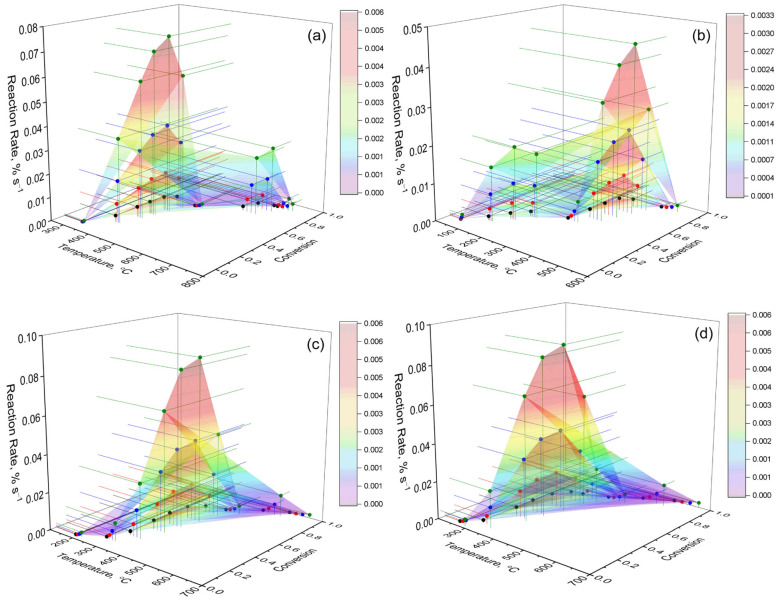
Three-dimensional surfaces of the dependence of the reaction rate (d*α*/d*T*) on temperature (*T*) and conversion rate (*α*) for: (**a**) branched p-PGM:AA:[CPDT] (10 mM); (**b**) branched p-PGM:AA:[CPDT] (80 mM); (**c**) cross-linked p-PGM:AA (without CPDT); (**d**) cross-linked p-PGM:AA:[CPDT] (80 mM).

The three-dimensional surfaces *R*(α,T) shown in Figure 15a–d reflect the distribution of reaction activity in the “temperature–conversion degree –reaction rate’ coordinates. For the branched copolymer at 10 mM CPDT (Figure 15a), a broad reaction zone is observed (α ≈ 0.2–0.8), indicating a distributed nature of the degradation processes. An increase in CPDT concentration to 80 mM (Figure 15b) is accompanied by localization of the maximum in the region of α ≈ 0.3–0.6, which may indicate partial overlap or localization of degradation processes.

The cross-linked copolymer without CPDT (Figure 15c) is characterized by a narrow, symmetrical kinetic surface maximum, indicating a more uniform nature of cross-linked structure degradation. The introduction of CPDT (80 mM) is accompanied by an expansion of the reaction zone and an increase in the heterogeneity of the rate distribution (Figure 15d). Thus, the formation of a cross-linked structure leads to localization of the region of maximum reaction activity on the *R*(α,T) surface, reflecting a change in the spatial distribution of kinetic processes.

**Figure 16:**
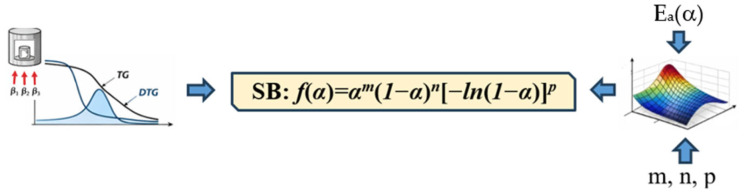
TG → Šesták–Berggren → three-dimensional surfaces *R*(*α*, *T*): integrated kinetic approach to thermal destruction analysis.

The asymmetry of the kinetic surfaces and the non-uniform distribution of reaction activity with respect to the degree of conversion suggest a complex thermolysis behavior that cannot be described by simple first-order or *n*th order kinetic models. The Šesták–Berggren function (Figure 16) was used to quantitatively describe the observed kinetic heterogeneity, which allows taking into account the combined contribution of autocatalytic and decaying stages within a single kinetic dependence.

**Figure 17:**
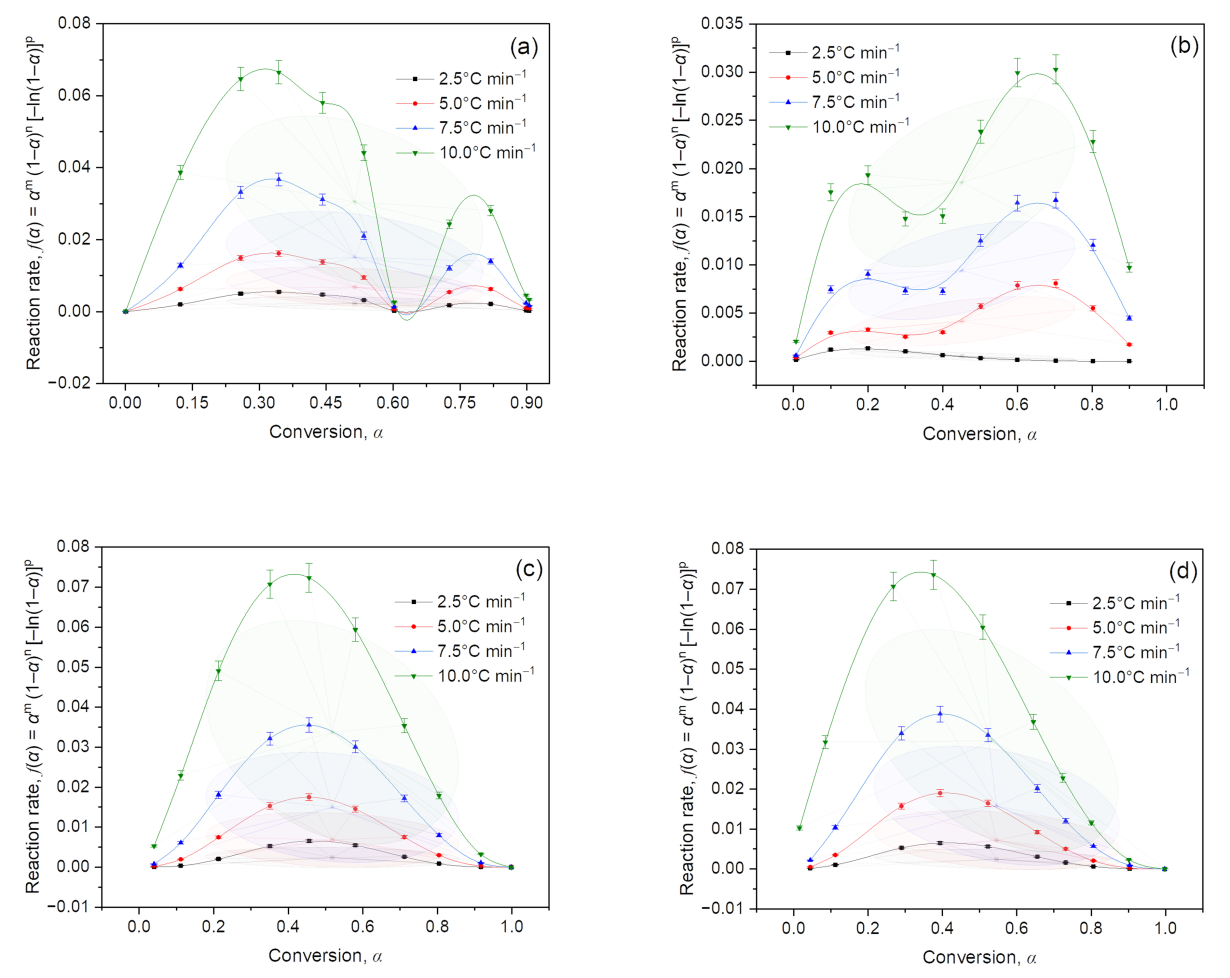
Dependence of the d*α*/d*t* reaction rate on the conversion degree *α* at different heating rates (2.5, 5, 7.5 and 10.0 °C·min^−1^). The shaded regions indicate changes in kinetic parameters in the conversion range for (**a**) branched p-PGM:AA:[CPDT] (10 mM); (**b**) branched p-PGM:AA:[CPDT] (80 mM); (**c**) cross-linked p-PGM:AA (without CPDT); (**d**) cross-linked p-PGM:AA:[CPDT] (80 mM).

The dependence of the reduced reaction rate *f*(*α*) = (d*α*/d*t*)/*β* on the α conversion for branched and cross-linked copolymers is shown in Figure 17a–d.

For the branched p-PGM:AA:[CPDT] copolymer (10 mM) (Figure 17a), the reaction rate maximum is observed at *α* ≈ 0.30–0.40; the asymmetry of the curves is likely associated with partial overlap of individual decomposition stages. With an increase in RAFT agent concentration to 80 mM (Figure 17b), the maximum shifts to the region of *α* ≈ 0.45–0.60, and the response amplitude increases. This may be attributed to an increase in the effective energy barrier of the initial stage and enhanced kinetic heterogeneity of the system.

The cross-linked copolymer without CPDT (Figure 17c) exhibits a smoother *f*(α) dependence with a broad maximum at *α* ≈ 0.35–0.50 and similar curve shapes at different *β*, which is consistent with a more homogeneous nature of thermal decomposition. The introduction of CPDT (80 mM) (Figure 17d) leads to an intensification of the peak and its shift towards higher values of *α*, whilst the overall shape of the curves is preserved.

An increase in heating rate is accompanied by an increase in the maximum reaction rate; however, the shape of the *f*(α) distribution is governed by the macromolecular organization of the copolymers: branched systems exhibit pronounced asymmetry and shifted maxima, whereas cross-linked structures demonstrate a more stable kinetic profile.

The kinetic parameters of the thermal decomposition of p-PGM:AA copolymers, calculated using non-linear parametric modeling (NPK) and the Šesták–Berggren (S-B) methods [39,40], are given in Table 7. The values of the effective activation energy *E_α_*, the pre-exponential factor ln*A*, and the parameters of the autocatalytic function *f*(*α*) = *A*·*α^m^*(1 − *α*)*^n^* have been determined.

For the branched copolymer at CPDT = 10 mM (sample a), the *E_α_* values obtained by NPK [14,18] and Š-B methods [39,40] (202.8 and 204.4 kJ·mol^−1^ respectively) are well consistent with each other. An increase in CPDT concentration to 80 mM (sample b) is accompanied by an increase in *E_α_* to 216.2 kJ·mol^−1^ (NPK) and 238.1 kJ·mol^−1^ (Š-B), as well as an increase in ln*A*, which may indicate enhanced kinetic heterogeneity of the system.

For cross-linked copolymers (samples c and d), the *E_α_* values calculated using the NPC method are in the narrow range of 201–204 kJ·mol^−1^, while the Š-B model yields higher values (259–277 kJ·mol^−1^), which is associated with different sensitivity of the methods to the shape of kinetic curves.

The parameters of the autocatalytic function also vary: branched systems are characterized by higher values of *n* (≈4.6–5.0), indicating a pronounced inhibitory stage at high conversion rates, whereas for cross-linked copolymers, more balanced values of *m* and *n* (*m* ≈ 1.4–1.6; *n* ≈ 2.3–2.5) are observed.

A comparison of isoconversional methods (FR, OFW) with model calculations shows that the kinetic profile of thermolysis is related to the macromolecular organization of the copolymers. For a branched copolymer at 10 mM, *E_α_* in FR decreases from 207.6 to 184.1 kJ·mol^−1^ with α growth, which reflects a change in the controlling stage of decomposition, while integral methods give close averaged values. At 80 mM, the initial *E_α_* values are significantly higher (329–370 kJ·mol^−1^), but as *α* increases, they decrease to ~kJ·mol^−1^, confirming the multi-stage nature of the process.

Overall, branched systems synthesized at higher RAFT agent concentrations are characterized by a broader *E_a_* variation range and increased kinetic heterogeneity, whereas cross-linked copolymers exhibit a more stable thermolysis profile. The detailed results of the statistical analysis (Student’s t-test and one-way ANOVA) are provided in the Supplementary Materials (Table S1).

The contour lines and color gradients in Figure 15 reflect the intensity of the process: the transition from the blue region to the yellow-red corresponds to an increase in the values of the kinetic parameter. The range of values varies from ~0.006–0.011 to ~0.065–0.076 depending on the type of copolymer structure.

**Figure 18:**
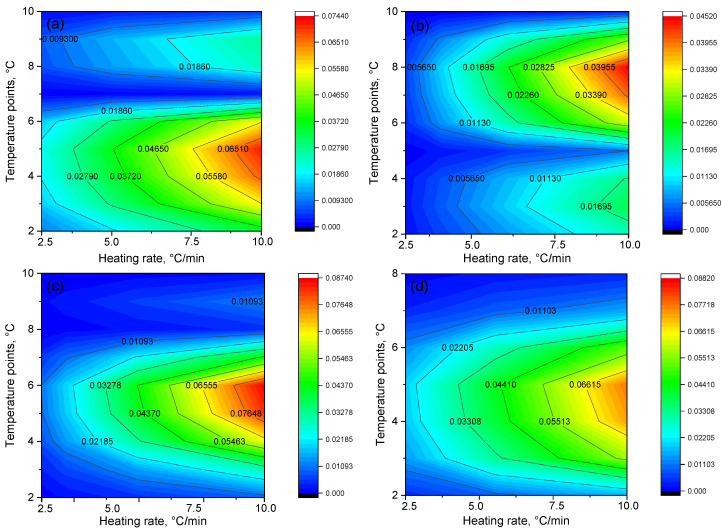
Contour maps of the reaction rate distribution in temperature/conversion coordinates for: (**a**) branched p-PGM:AA:[CPDT] (10 mM); (**b**) branched p-PGM:AA: [CPDT] (80 mM); (**c**) cross-linked p-PGM:AA (without CPDT); (**d**) cross-linked p-PGM:AA: [CPDT] (80 mM). The obtained dependencies indicate a more heterogeneous thermolysis behavior for RAFT-modified copolymers.

For a branched copolymer at 10 mM CPDT (Figure 18a), a pronounced gradient is recorded with an increase in the parameter from ~0.009 to ~0.065, shifted to the region of higher temperatures and conversion rates. With an increase in CPDT concentration to 80 mM (Figure 18b), the range of values narrows (~0.006–0.040), and the maximum becomes localized, which may reflect a change in the distribution of effective energy barriers.

The cross-linked copolymer without CPDT (Figure 18c) is characterized by the highest parameter values (up to ~0.076) and the most intense gradient. The introduction of CPDT (80 mM) (Figure 18d) is accompanied by smoothing of distribution and reduction of kinetic surface contrast.

The range of parameter variation is associated with the macromolecular organization of the copolymers and the concentration of the RAFT agent: the highest values are characteristic of cross-linked structures without CPDT, whereas branched systems synthesized in the presence of the RAFT agent exhibit a narrower range and less pronounced maxima. These results are consistent with the observed *E_a_*(*α*) relationships.

The spatial distribution of the kinetic parameter in the “temperature-conversion rate” coordinates allows to identify areas of increased reaction activity that are critical for assessing the thermal stability of copolymers. The spatial distribution of the kinetic parameter in the “temperature–conversion rate” coordinates allows identification of regions of increased reaction activity that are critical for assessing the thermal stability of copolymers. For the cross-linked copolymer without CPDT, more localized maxima are observed, reflecting a concentrated nature of degradation, whereas branched systems synthesized in the presence of the RAFT agent are characterized by a broader and more heterogeneous distribution of reaction activity. This may indicate a wider temperature range of thermolysis and increased kinetic heterogeneity of the branched structures.

As a result, branched systems require temperature control in a wider heating range, while for cross-linked copolymers the critical region is limited to a narrower range. Kinetic parameter distribution maps can be used as a quantitative tool to identify temperature zones of increased risk of accelerated degradation during processing and operation of p-PGM: AA copolymers.

## 4. Conclusions

The influence of 2-cyano-2-propyl dodecyl trithiocarbamate (CPDT) on the properties of p-PGM:AA copolymers was investigated using a combination of ^1^H/^13^C NMR spectroscopy, gel permeation chromatography (GPC), transmission electron microscopy (TEM), thermal analysis, mass spectrometry, kinetic modeling, and quantum-chemical calculations.

An increase in CPDT concentration was accompanied by a decrease in the relative intensity of the vinyl proton signals associated with maleinate fragments in the ^1^H NMR spectra, consistent with their progressive consumption during copolymerization. GPC analysis of the soluble fraction revealed changes in the molecular weight characteristics and a decrease in dispersity with increasing CPDT content.

Alkaline hydrolysis followed by ^1^H NMR and GPC analysis confirmed cleavage of the polyester component, yielding low-molecular-weight hydrolysis products (*M_n_* = 1370 g·mol^−1^, *Đ* = 1.035). These results provide additional information on the composition of the soluble structural fragments present in the copolymer system.

TEM analysis revealed differences in domain size distributions between samples synthesized in the absence and in the presence of CPDT. Samples containing CPDT exhibited an increase in the average equivalent domain diameter together with a broader size distribution.

According to TG/DTG analysis, the presence of CPDT was associated with the appearance of an additional low-temperature degradation stage. TG–MS results indicated changes in the composition of the evolved volatile products compared with samples prepared in the absence of the RAFT agent.

Quantum-chemical calculations showed that incorporation of RAFT-derived fragments modifies the electronic characteristics of the model structures, including a reduction in the HOMO–LUMO energy gap. The calculated results are consistent with the observed thermal behavior of the copolymers.

Isoconversional and nonlinear kinetic analyses revealed differences in the thermal degradation behavior of branched and cross-linked copolymers. The observed variations depend on CPDT concentration and are consistent with differences in the structure and composition of the investigated materials.

Overall, the results demonstrate that the presence of CPDT influences the structure, molecular weight characteristics, morphology, and thermal behavior of p-PGM:AA copolymers. The obtained data are consistent with the participation of RAFT-mediated processes in determining the properties of the resulting materials.

## Figures and Tables

**Table 1:** Semi-quantitative structural descriptors of branched p-PGM:AA:[CPDT] copolymers synthesized at different CPDT concentrations.

[CPDT] × 10^−2^, M	BI*	RI*
1.00	1.0186	0.0120
3.00	0.6009	0.0236
5.00	1.7467	0.0199
8.00	1.2209	0.0675

**Table 2:** Molecular weight characteristics and dispersity of p-PGM:AA:[CPDT] copolymers synthesized at different CPDT concentrations.

[CPDT] × 10^−2^, M	*M_n_* × 10^3^, g·mol^−1^	*M_w_* × 10^3^, g·mol^−1^	*M_z_* × 10^3^, g·mol^−1^	*Đ (M_w_*/*M_n_)*
1.00	9.68	12.20	20.60	1.26
3.00	3.78	4.36	6.20	1.15
5.00	3.51	4.17	6.89	1.19
8.00	3.29	3.75	5.10	1.14

**Table 3:** Average equivalent domain diameters (TEM) and previously reported swelling degrees of p-PGM:AA copolymers [9].

Sample	Average Equivalent Domain Diameter, nm	Swelling Degree, %
p-PGM:AA	18.6 ± 13.6	187.1
p-PGM:AA:[CPDT] = 80 mM	24.2 ± 15.7	385.1

**Table 4:** Thermal degradation characteristics of p-PGM:AA copolymers with different macromolecular architectures at *β* = 2.5 °C·min^−1^.

[CPDT] × 10^−2^, M	T_max_ (Main.)_,_ °C	${\left|\frac{dm}{dT}\right|}_{max}$, %·°C^−1^	ΔT_1/2,_ °C	$\int_{T_{1}}^{T_{2}}\left|\frac{dm}{dT}\right|dT,$ *%*	Δm, %	m_res_, %
branched copolymer						
1.0	354.0	0.5	23.0	13.0	44.3	5.9
8.0	370.0	0.7	20.0	16.0	42.4	3.7
cross-linked copolymer						
–	356.0	1.3	10.6	13.6	45.6	6.0
8.0	355.0	1.4	20.2	26.6	46.9	9.4

**Table 5:** Energy characteristics of the model systems calculated at the B3LYP/6-311(d,p) level of theory.

Parameter	Maleate	Maleate + RAFT
E_tot_, Hartree	−648.392	−2831.783
HOMO, Hartree	−0.294	−0.231
LUMO, Hartree	−0.195	−0.187
ΔE, Hartree	0.099	0.044
η, Hartree	0.050	0.022
σ, Hartree^−1^	10.070	22.820
IP, Hartree	0.294	0.231

**Table 6:** Activation energy (*E_a_*, kJ·mol^−1^) determined by Friedman and OFW methods at different degrees of α transformation. Data are mean ± standard deviation (*n* = 3).

Sample	Method	0.1	0.3	0.5	0.7	0.9	*Ē*_a_, kJ·mol^−1^)
Branched copolymer							
(a) 10 mM	FR	207.6 ± 0.9	193.8 ± 0.9	188.3 ± 0.9	184.1 ± 0.9	189.5 ± 0.9	189.5 ± 0.9
	OFW	233.4 ± 1.1	233.4 ± 1.1	217.9 ± 0.9	207.3 ± 0.9	213.5 ± 0.9	228.7 ± 1.1
(b) 80 mM	FR	329.2 ± 1.1	190.5 ± 0.9	190.5 ± 0.9	185.6 ± 0.9	188.3 ± 0.9	204.2 ± 0.9
	OFW	369.7 ± 1.3	214.2 ± 0.9	214.2 ± 0.9	209.0 ± 0.9	212.1 ± 0.9	229.8 ± 1.2
Cross-linked copolymer							
(c) −	FR	180.8 ± 0.8	184.0 ± 0.9	185.5 ± 0.9	186.4 ± 0.9	202.2 ± 0.9	189.2 ± 0.9
	OFW	203.6 ± 0.9	207.2 ± 0.9	209.0 ± 0.9	210.0 ± 1.0	228.0 ± 1.1	213.1 ± 0.9
(d) 80 mM	FR	186.1 ± 0.9	188.8 ± 0.9	189.7 ± 0.9	191.5 ± 0.9	191.5 ± 0.9	194.4 ± 0.9
	OFW	204.9 ± 0.9	208.0 ± 0.9	209.0 ± 0.9	229.5 ± 1.2	229.5 ± 1.2	214.1 ± 0.9

**Table 7:** Kinetic parameters of thermal decomposition of branched and cross-linked p-PGM:AA copolymers with different concentrations of CPDT *E_a_*—kJ·mol^−1^; *A*—s^−1^. Data are presented as mean ± SD (*n* = 3).

Sample	$E_{\alpha }^{NPM}\left(\alpha \right)$, kJ·mol^−1^	ln *A* (NPK)	*f*(*α*) = *A*·*α*^m^⋅(1 − *α*)^n^		$E_{\alpha }^{S-B}\left(\alpha \right)$ kJ·mol^−1^	ln *A* (Š-B)
			M	n		
Branched copolymer						
(a) p-PGM: AA: [CPDT] = 10 mM	202.8 ± 0.6	59.2 ± 0.02	2.3	4.6	204.4 ± 0.4	67.4 ± 0.02
(b) p-PGM: AA: [CPDT] = 80 mM	216.2 ± 0.4	118.6 ± 0.02	1.0	5.0	238.1 ± 0.5	149.4 ± 0.02
Cross-linked copolymer						
(c) p-PGM: AA	201.3 ± 0.9	40.1 ± 0.02	1.6	2.3	259.1 ± 0.6	99.1 ± 0.02
(d) p-PGM: AA: [CPDT] = 80 mM	204.3 ± 0.4	33.9 ± 0.02	1.4	2.5	276.5 ± 0.6	71.9 ± 0.02

## Data Availability

The original contributions presented in this study are included in the article/Supplementary Materials. Further inquiries can be directed to the corresponding authors.

## Supplementary Materials

The following supporting information can be downloaded at: https://www.mdpi.com/article/10.3390/polym18131599/s1. Supplementary Materials S1: Reaction Scheme of RAFT Copolymerization of Polypropylene Glycol Maleate with Acrylic Acid; Scheme S1. Radical copolymerization of polypropylene glycol maleate with acrylic acid in the presence of the RAFT agent CPDT leading to branched p-PGM:AA:[CPDT] copolymers. Supplementary Materials S2: ^1^H and ^13^C NMR Spectra of p-PGM:AA Copolymers; Figure S1. ^1^H and ^13^C NMR spectra of p-PGM:AA:CPDT copolymers synthesized in the presence of different concentrations of the RAFT agent CPDT. Supplementary Materials S3: FTIR Spectra of p-PGM and p-PGM:AA Copolymers; Figure S2. FTIR spectra of p-PGM and p-PGM:AA copolymers synthesized with different concentrations of the RAFT agent CPDT. Supplementary Materials S4: Raman Spectra of p-PGM:AA Copolymers; Figure S3. Raman spectra of p-PGM:AA (50:50) copolymers synthesized. Supplementary Materials S5: Thermal Evolution of the Morphology of p-PGM:AA Copolymers; Figure S4. SEM micrographs of the synthesized copolymers. Supplementary Materials S6: Statistical Analysis of Activation Energy for Copolymer Compositions; Table S1. Statistical analysis of activation energy for different copolymer compositions.

## Abbreviations

The following abbreviations are used in this manuscript:

RAFT	Reversible Addition–Fragmentation Chain Transfer
CPDT	2-Cyano-2-propyl dodecyl trithiocarbonate
p-PGM	Polypropylene glycol maleate
AA	Acrylic acid
TG	Thermogravimetric analysis
DTG	Differential thermogravimetry
TG–MS	Thermogravimetry–mass spectrometry
TP-MS	Temperature-programmed mass spectrometry
SEM	Scanning electron microscopy
TEM	Transmission electron microscopy
NMR	Nuclear magnetic resonance
^1^H NMR	Proton nuclear magnetic resonance
^13^C NMR	Carbon-13 nuclear magnetic resonance
HPLC	High-performance liquid chromatography
IR	Infrared spectroscopy
DFT	Density Functional Theory
PM6	Parametric Method 6
B3LYP	Becke, 3-parameter, Lee–Yang–Parr functional
GPC	Gel permeation chromatography
FR	Friedman method
OFW	Ozawa–Flynn–Wall method
NPK	Non-parametric kinetics
SVD	Singular value decomposition
ANOVA	Analysis of variance
HOMO	Highest occupied molecular orbital
LUMO	Lowest unoccupied molecular orbital
