# Supplementary material for: Architecture-Dependent Thermal Decomposition of RAFT-Modified Polypropylene Glycol Maleate-Acrylic Acid Copolymers: Results of TG–MS and Kinetic Analysis
Source: Polymers (Basel). 2026 Jun 26;18(13):1599. doi: 10.3390/polym18131599 (PMC13364207; doi:10.3390/polym18131599)
Supplement: Supplementary file 1 [file polymers-18-01599-s001.zip › polymers-4303518-supplementary.pdf]

Supplementary Materials S1

Reaction Scheme of RAFT Copolymerization of Polypropylene Glycol Maleate with Acrylic Acid

A) Initiation

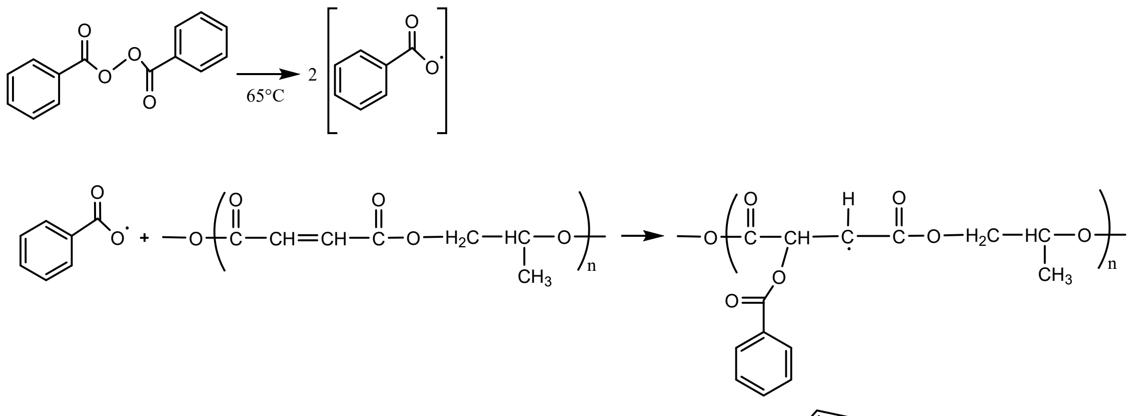

B) Propagation

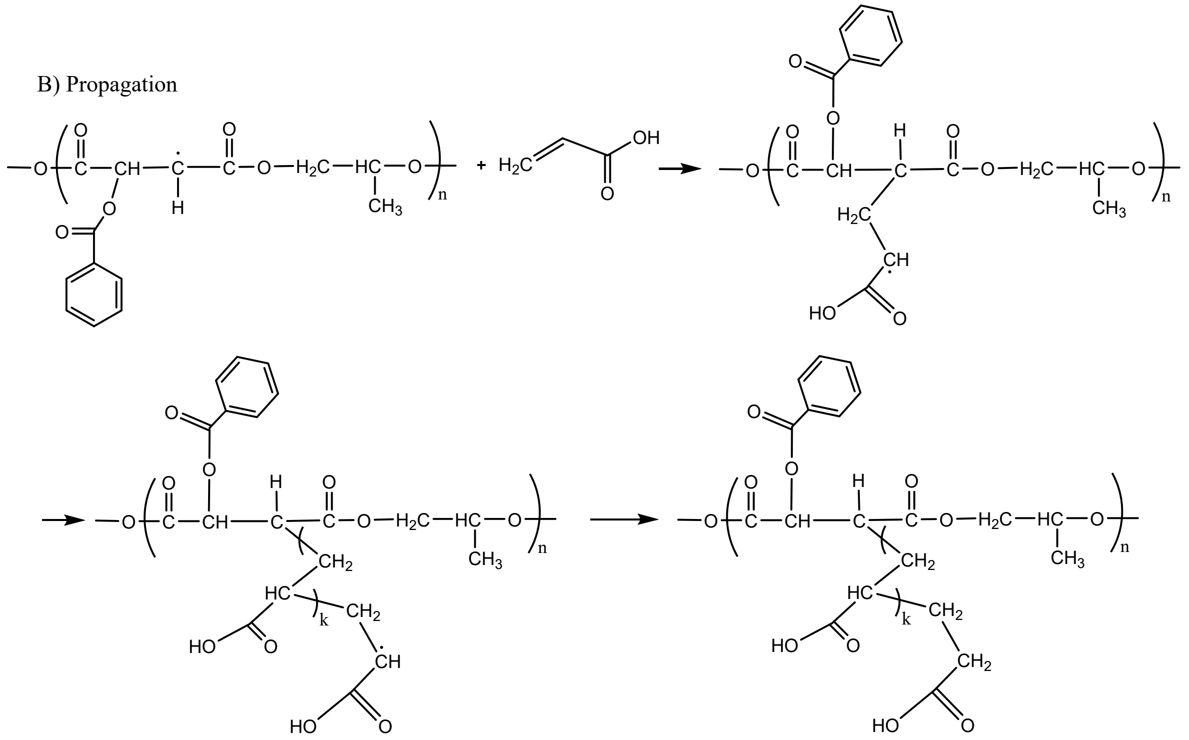

C) Addition to RAFT agent

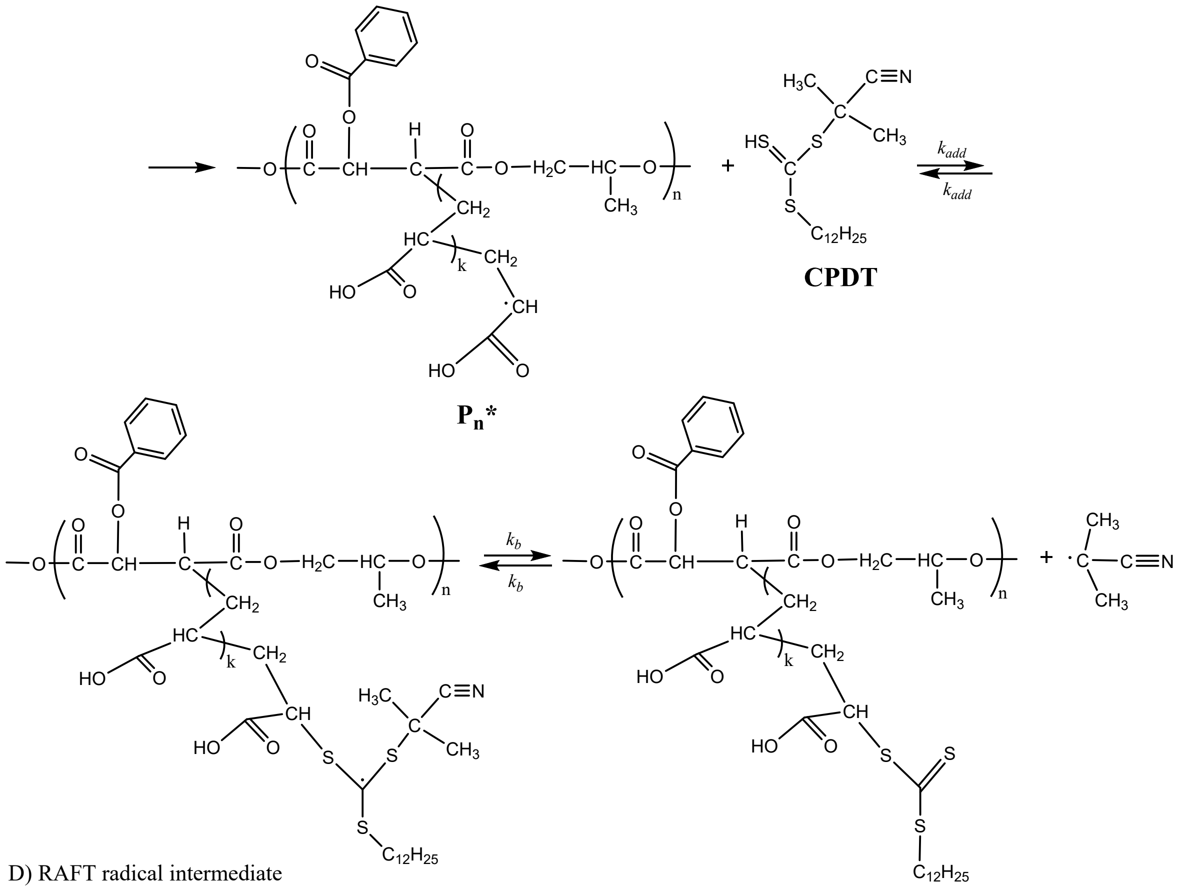

D) RAFT radical intermediate

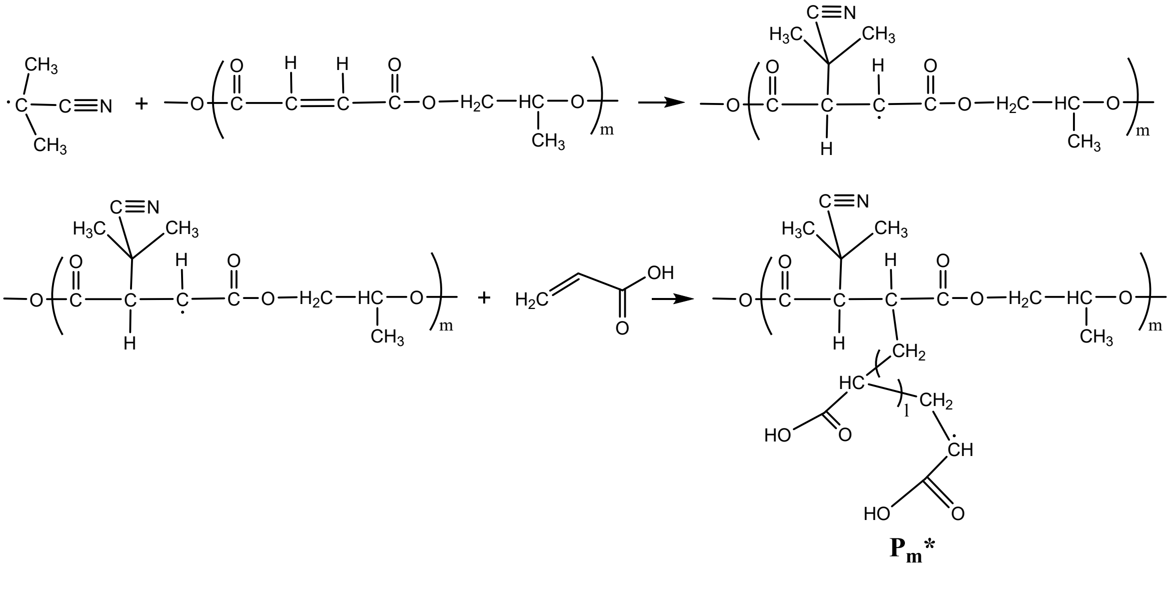

E) Dynamic equilibrium

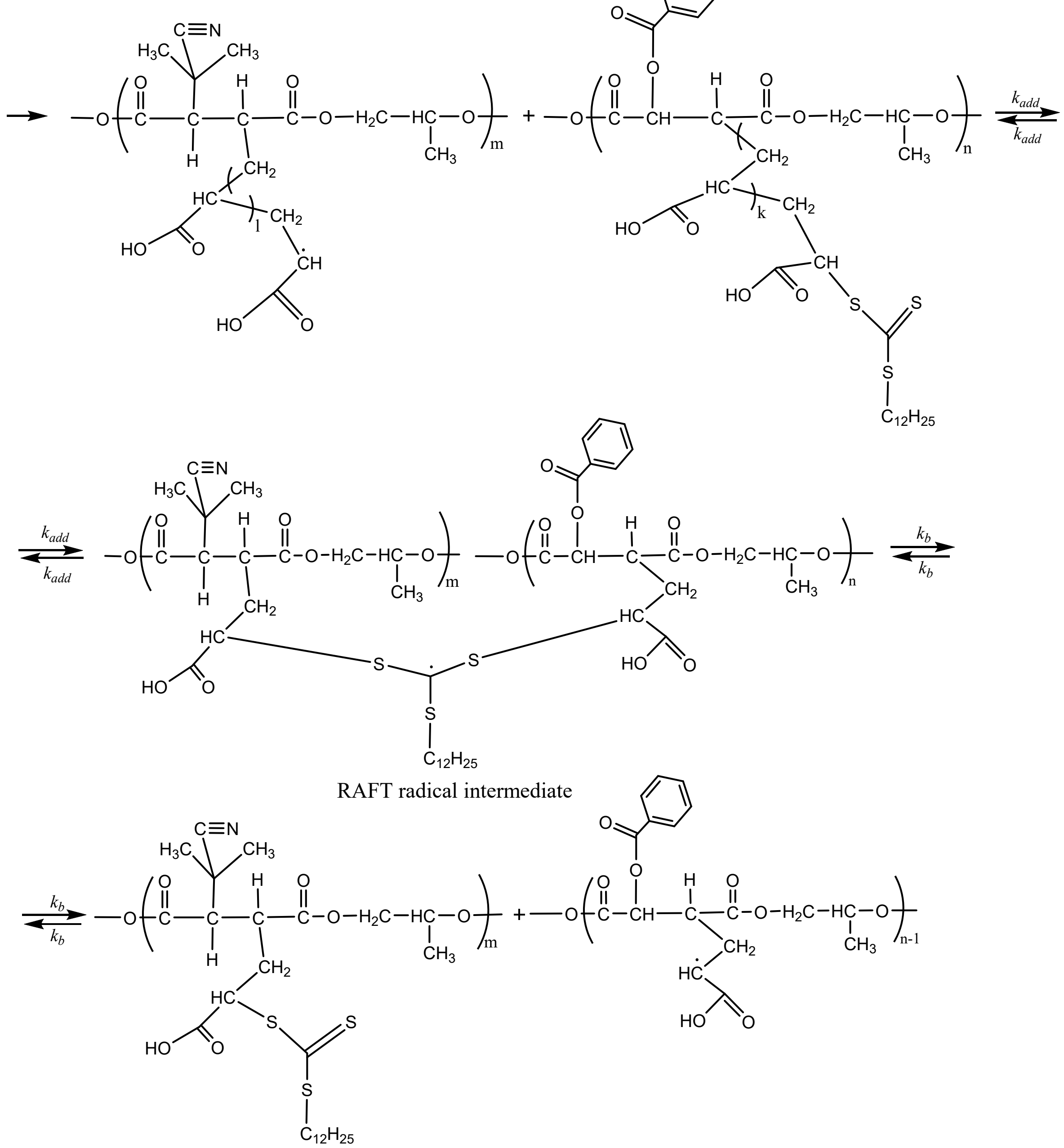

Scheme S1. Radical copolymerization of polypropylene glycol maleate with acrylic acid in the presence of the RAFT agent CPDT leading to branched p-PGM:AA:[CPDT] copolymers.

Supplementary Materials S2

^{1}H and ^{13}C NMR Spectra of p-PGM:AA:CPDT Copolymers

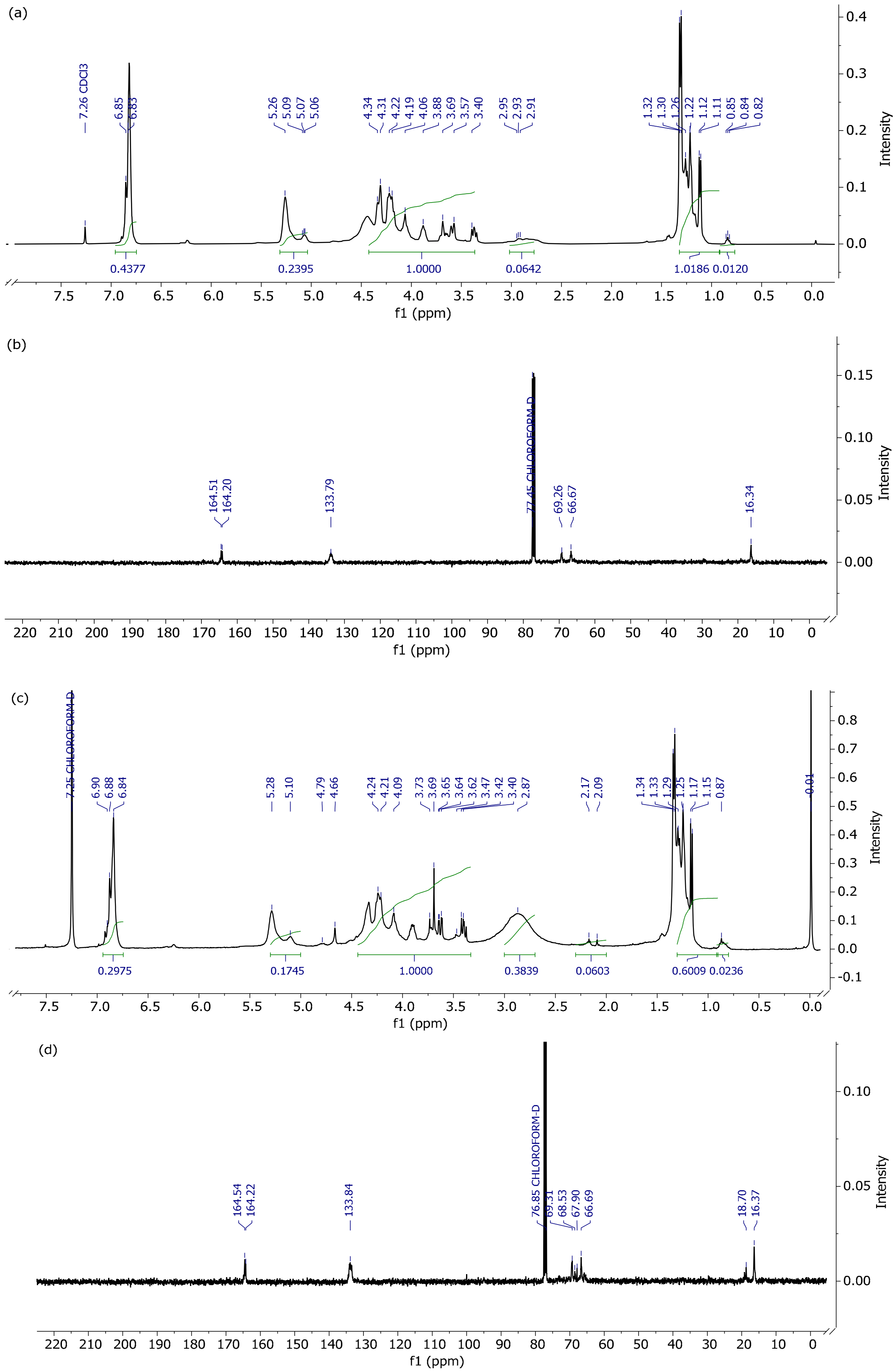

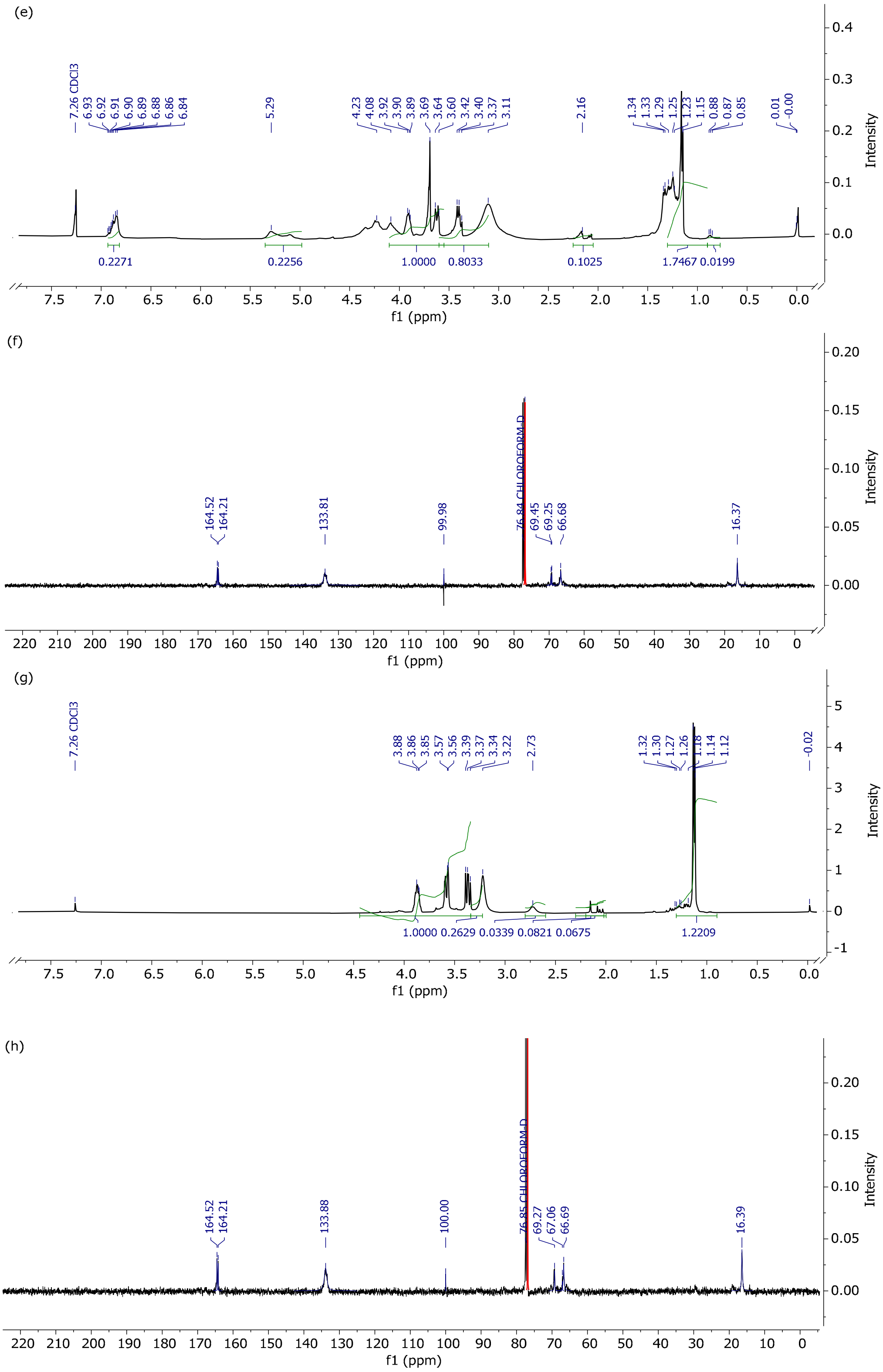

Figure S1. ^{1}H and ^{13}C NMR spectra of p-PGM:AA:CPDT copolymers synthesized in the presence of different concentrations of the RAFT agent CPDT:

(a) ^{1}H NMR spectrum, [CPDT] = 10 mM;

(b) ^{13}C NMR spectrum, [CPDT] = 10 mM;

(c) ^{1}H NMR spectrum, [CPDT] = 30 mM;

(d) ^{13}C NMR spectrum, [CPDT] = 30 mM;

(e) ^{1}H NMR spectrum, [CPDT] = 50 mM;

(f) ^{13}C NMR spectrum, [CPDT] = 50 mM;

(g) ^{1}H NMR spectrum, [CPDT] = 80 mM;

(h) ^{13}C NMR spectrum, [CPDT] = 80 mM.

Supplementary Materials S3

FTIR Spectra of p-PGM and p-PGM:AA Copolymers

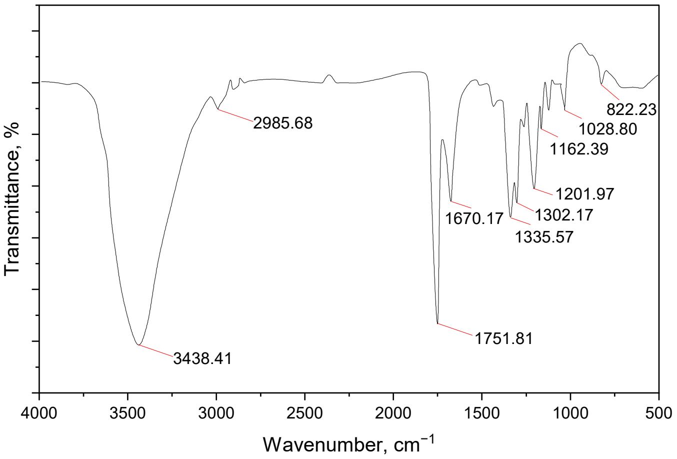

(a)

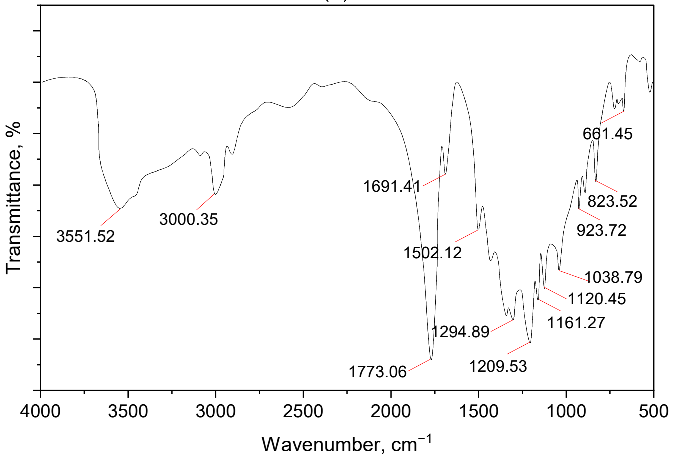

(b)

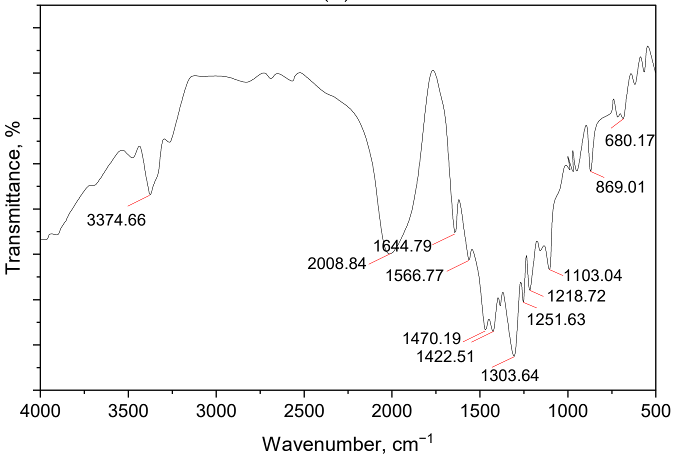

(c)

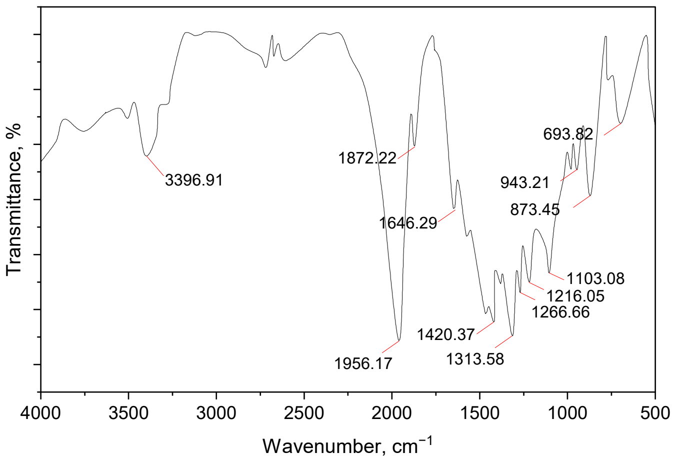

(d)

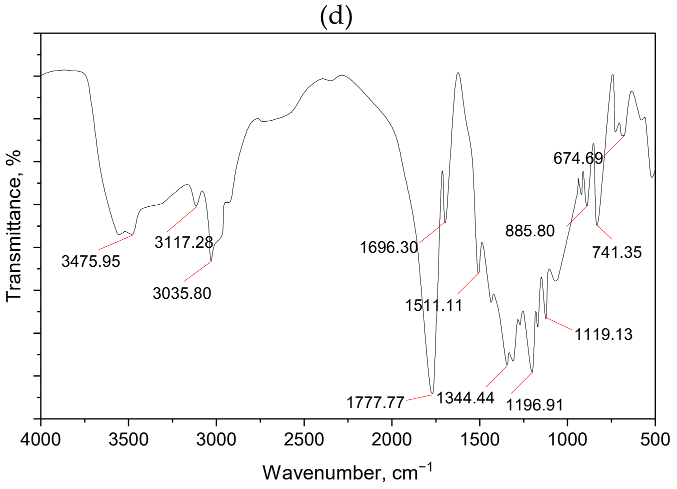

(e)

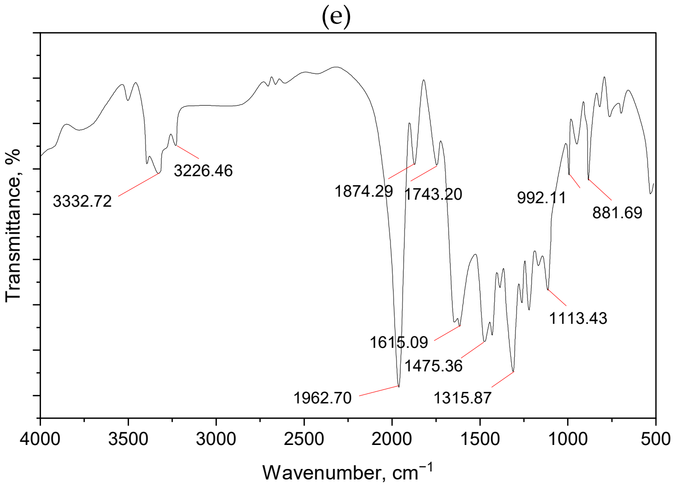

(f)

Figure S2. FTIR spectra of p-PGM and p-PGM:AA copolymers synthesized with different concentrations of the RAFT agent CPDT:
(a) p-PGM;
(b) p-PGM:AA;
(c) p-PGM:AA synthesized in the presence of CPDT ([CPDT] = 10 mM);
(d) p-PGM:AA synthesized in the presence of CPDT ([CPDT] = 30 mM);
(e) p-PGM:AA synthesized in the presence of CPDT ([CPDT] = 50 mM);
(f) p-PGM:AA synthesized in the presence of CPDT ([CPDT] = 80 mM)

Supplementary Materials S4

Raman Spectra of p-PGM:AA Copolymers

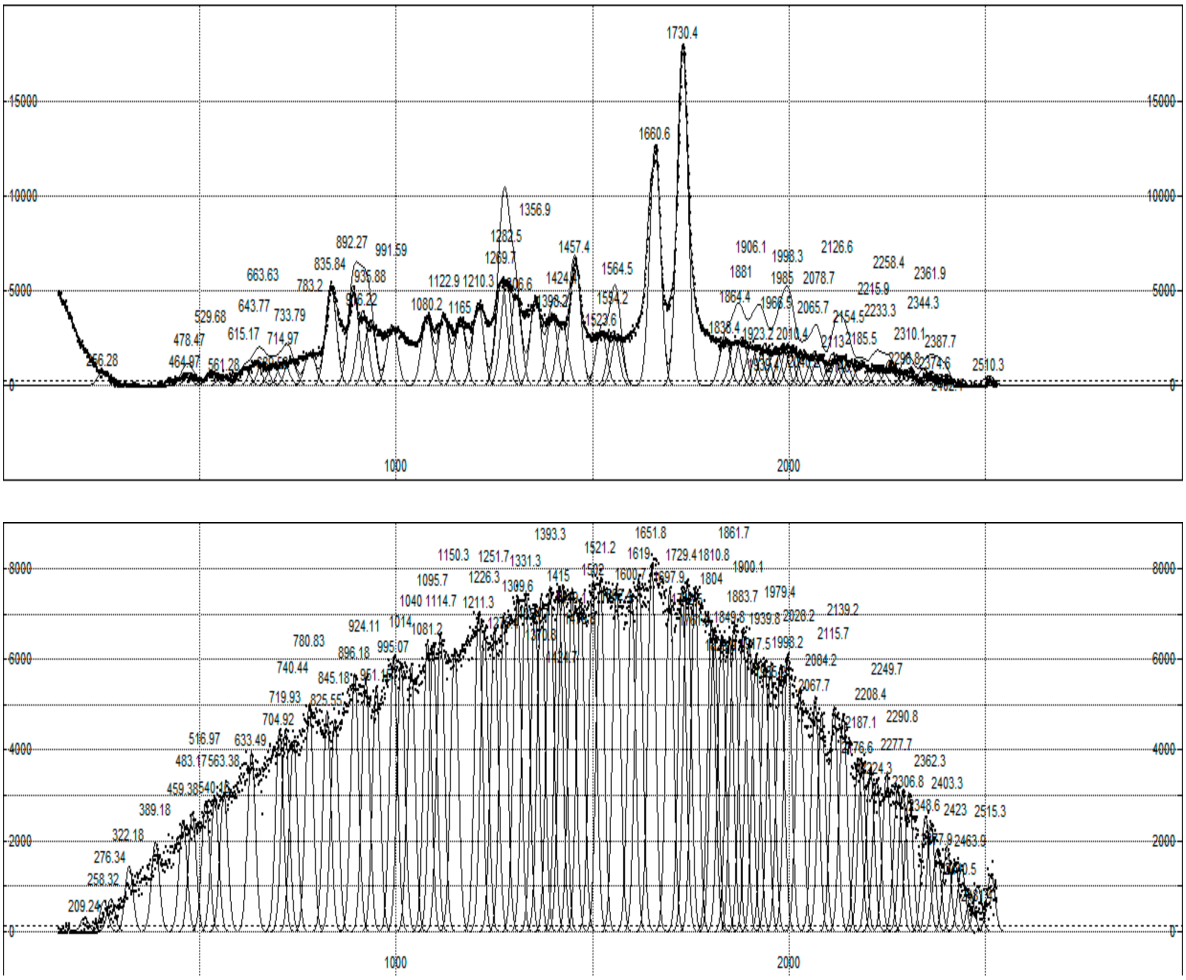

(a)

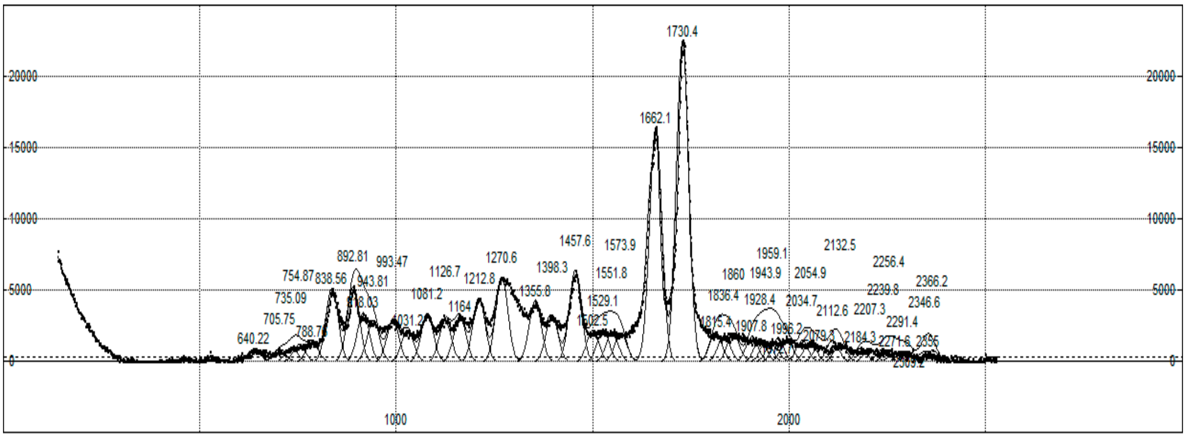

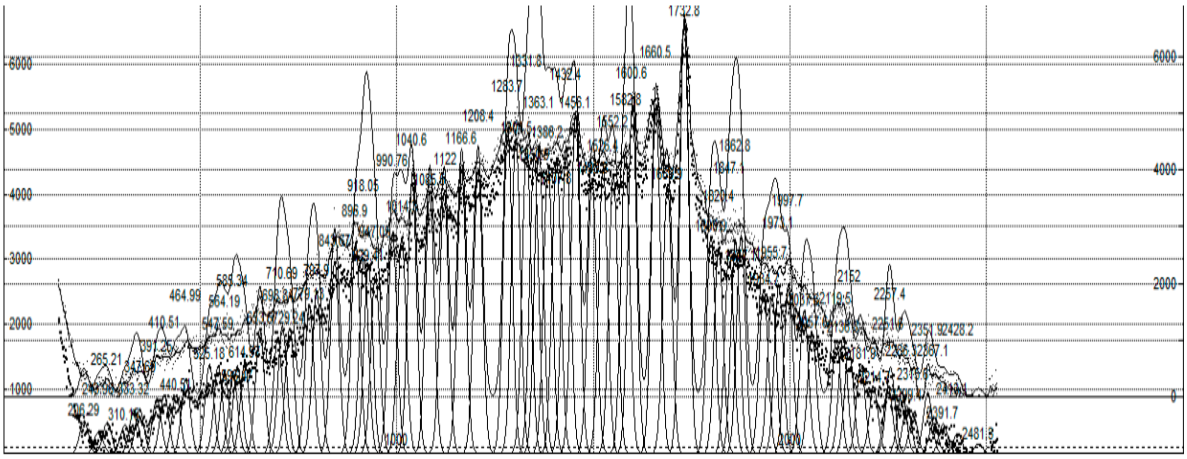

(b)

Figure S3. Raman spectra of p-PGM:AA (50:50) copolymers synthesized:
(a) in the absence of the RAFT agent; (b) in the presence of CPDT ([CPDT] = 50 mM)

Supplementary Materials S5

Thermal Evolution of the Morphology of p-PGM:AA Copolymers

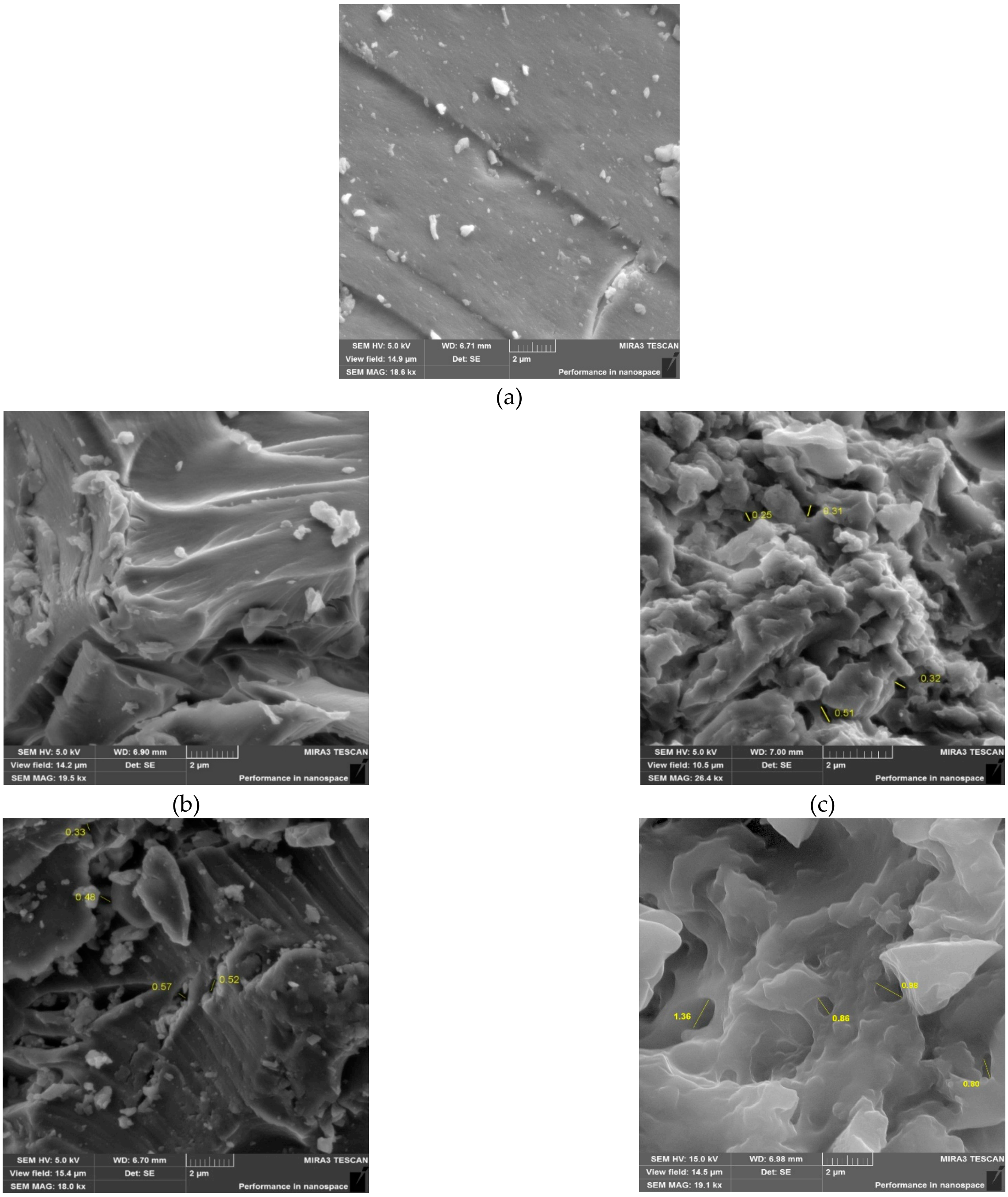

(a)

(b) (c)

(d) (e)

Figure S4. SEM micrographs of the synthesized copolymers:

(a) p-PGM:AA;

(b) p-PGM:AA synthesized in the presence of CPDT ([CPDT] = 10 mM);

(c) p-PGM:AA synthesized in the presence of CPDT ([CPDT] = 30 mM);

(d) p-PGM:AA synthesized in the presence of CPDT ([CPDT] = 50 mM);

(e) p-PGM:AA synthesized in the presence of CPDT ([CPDT] = 80 mM).

Supplementary Materials S6

Statistical Analysis of Activation Energy for Copolymer Compositions

Table S1. Statistical analysis of activation energy for different copolymer compositions

(a) One-way ANOVA results

**Composition**	***F*-statistic**	***p*-value**
(a–d)	373.61	6.19×10^{-9}

(b) Pairwise t-test results

**Composition**	***p*-value**	**Interpretation of Results**
a vs b	1.7×10^{-5}	$p < 0.05$
a vs c	0.039	$p < 0.05$
a vs d	0.003	$p < 0.05$
b vs c	1.1×10^{-5}	$p < 0.05$
b vs d	2.4×10^{-5}	$p < 0.05$
c vs d	0.012	$p < 0.05$

Note: One-way ANOVA was used to evaluate statistical differences among copolymer compositions (a–d). Pairwise comparisons were performed using a two-tailed Student's t-test. Differences were considered statistically significant at $p < 0.05$.
